# Photoelectrocatalytic Hydrogen Generation: Current Advances in Materials and *Operando* Characterization

**DOI:** 10.1002/gch2.202400011

**Published:** 2024-07-04

**Authors:** Mohammed Ahmed Zabara, Burak Ölmez, Merve Buldu‐Akturk, Begüm Yarar Kaplan, Ahmet Can Kırlıoğlu, Selmiye Alkan Gürsel, Mihrimah Ozkan, Cengiz Sinan Ozkan, Alp Yürüm

**Affiliations:** ^1^ Sabanci University SUNUM Nanotechnology Research Center Istanbul 34956 Türkiye; ^2^ Faculty of Engineering and Natural Sciences Sabanci University Istanbul 34956 Türkiye; ^3^ Department of Electrical and Computer Engineering University of California Riverside CA 02521 USA; ^4^ Department of Mechanical Engineering University of California Riverside CA 02521 USA

**Keywords:** bandgap, charge recombination, green hydrogen, heterojunction, operando characterization, photoelectrocatalysis

## Abstract

Photoelectrochemical (PEC) hydrogen generation is a promising technology for green hydrogen production yet faces difficulties in achieving stability and efficiency. The scientific community is pushing toward the development of new electrode materials and a better understanding of the underlying reactions and degradation mechanisms. Advances in photocatalytic materials are being pursued through the development of heterojunctions, tailored crystal nanostructures, doping, and modification of solid‐solid and solid‐electrolyte interfaces. *Operando* and in situ techniques are utilized to deconvolute the charge transfer mechanisms and degradation pathways. In this review, both materials development and *Operando* characterization are covered for advancing PEC technologies. The recent advances made in the PEC materials are first reviewed including the applied improvement strategies for transition metal oxides, nitrites, chalcogenides, Si, and group III‐V semiconductor materials. The efficiency, stability, scalability, and electrical conductivity of the aforementioned materials along with the improvement strategies are compared. Next, the *Operando* characterization methods and cite selected studies applied for PEC electrodes are described. *Operando* studies are very successful in elucidating the reaction mechanisms, degradation pathways, and charge transfer phenomena in PEC electrodes. Finally, the standing challenges and the potential opportunities are discussed by providing recommendations for designing more efficient and electrochemically stable PEC electrodes.

## Graphical abstract

Green hydrogen is crucial for reducing CO_2_ emissions, with photoelectrochemical (PEC) production using solar energy to split water as a promising method. Key challenges include durability, efficiency, and cost. Advances in novel materials and Operando techniques are essential for improving PEC systems, unlocking their potential for a sustainable, low‐carbon future.

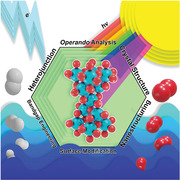

## 1 Introduction

Green hydrogen usage holds significant importance in addressing the challenge for reducing CO_2_ emissions, where photoelectrochemical (PEC) green hydrogen production emerges as a promising solution. This method utilizes solar energy to split water into hydrogen and oxygen, offering a clean and renewable source of fuel. However, there are challenges related to durability, efficiency, and cost that must be addressed for practical utilization and widespread adoption. To overcome these obstacles, novel material development is crucial; by exploring new materials with improved stability, conductivity, and catalytic properties, one can enhance the performance and durability of PEC systems. Additionally, the development of Operando techniques has become essential which enables real‐time monitoring and analyses of materials and interfaces during hydrogen production, facilitating the optimization of processes for practical applications. By focusing on materials advancements and Operando techniques, we discuss the current limitations and describe pathways to unlock the full potential of PEC green hydrogen production that can lead to a disruptive transition for a sustainable energy and low‐carbon future.

Today, sustainability and energy security are amongst the most important socio‐economic issues in the world. Especially, the use of fossil fuels causes raise in total net CO_2_ emissions and that results in dramatic changes in our world such as the increment in the temperature of the Earth's surface and eventually affecting the average rate of sea levels, by a 20 cm rise from 1901 to 2018, and have induced significant adverse effects to ecosystem diversity.^[^ ^1^, ^2^ ^]^ Today, hydrogen is being considered as a very promising alternative to fossil fuels due to its high energy content (≈142 kJ mol^−1^) which is 2.5 times higher than that of fossil fuels, and its cleanliness which produces water when burned.^[^ ^3^, ^4^ ^]^ Although hydrogen is attractive as a clean fuel or energy carrier, 90% of the world's hydrogen is currently produced from fossil fuel‐based sources. Therefore, the production of green hydrogen, which is produced by using renewable power sources with a low carbon footprint, is crucial to achieving the clean energy targets of countries. The interest in green hydrogen increased gradually due to the carbon‐neutral energy ecosystem targets of several countries including Japan,^[^ ^5^ ^]^ the European Union (EU) countries^[^ ^6^ ^]^ and the USA,^[^ ^7^ ^]^ that launched multi‐year initiatives and projects to establish roadmaps and encourage the production of clean H_2_ and the use of H_2_‐based technologies in various applications. The US Strategy and Roadmap calls for collaboration among federal agencies, industry, academia, local and Tribal communities, environmental and justice groups, labor unions, and other stakeholders to accelerate hydrogen market adoption. It establishes concrete targets, market‐driven metrics, and actions to measure success across sectors. At the Paris Climate Conference COP21 in 2015, parties of the United Nations Framework Convention on Climate Change (UNFCCC) reached an agreement to accelerate actions to mitigate climate change, after which growth in the hydrogen economy has become more important and essential to minimize carbon footprint.^[^ ^8^ ^]^

The Japanese government aims for a carbon‐neutral future, emphasizing energy security by establishing an international hydrogen supply chain and promoting the use of ammonia in thermal power generation by 2030. This briefing examines Japan's hydrogen strategy, policy initiatives, and research efforts, including transportation technologies, while also addressing the challenges in integrating hydrogen into the country's energy system. Clean hydrogen, produced with renewable electricity, is not yet as cost‐competitive as hydrogen from natural gas. However, studies suggest that an EU energy system with significant hydrogen and renewable gases would be more cost‐effective than one relying heavily on electrification. The EU prioritizes hydrogen research and innovation, funding projects and accelerating development via the EU hydrogen strategy and the European Clean Hydrogen Alliance. Almost all EU Member States include hydrogen in their 2021–2030 energy and climate plans, focusing on its role in transport and industry.

In the last few decades, interest in hydrogen production by water splitting has been growing rapidly through developing technologies based on electrolysis,^[^ ^9^ ^]^ photolysis,^[^ ^10^ ^]^ and photoelectrolysis.^[^ ^11^ ^]^ Among the renewable resources, the use of solar energy is very attractive due to the unconfined supply of power necessary for H_2_ production. Photoelectrolysis (PEC) is a process by which water is decomposed into H_2_ and O_2_ gasses under light‐generated charge carriers and applied potential bias.^[^ ^12^ ^]^ PEC can become a promising technology for H_2_ production which can replace fossil fuels since it is one of the simplest and cleanest methods.^[^ ^13^ ^]^ Moreover, PEC is a promising H_2_ production pathway due to the use of low‐cost semiconductor materials to obtain reasonable conversion efficiency at low temperatures.^[^ ^14^ ^]^ The theoretical solar‐to‐hydrogen efficiency for a PEC system is ≈10% and the estimated cost for such efficiency amounts to roughly 8.4 $/kgH_2_.^[^ ^15^ ^]^ In the EU there are great examples for demo production using PEC: Spanish energy companies Repsol and Enagas have developed technologies for H_2_ production via PEC.^[^ ^16^ ^]^ In southeast Asia, South Korean scientists have developed a PEC system that includes a perovskite photocathode and lignocellulosic biomass.^[^ ^17^ ^]^ The researchers used a Nafion membrane and an organic‐inorganic halide perovskite absorber in their photoelectrochemical cell, opting for lignocellulosic biomass over water as an electron source due to its lower required potential. This approach not only addresses the challenges of water oxidation but also produces valuable chemicals like vanillin and acetovanillone while preserving cellulose for further use. Another successful example is Syzygy Plasmonics, a Rice University (USA) technology startup, has secured $76 million in Series C funding, marking one of the largest rounds for a venture originating from a Rice lab. The company aims to commercialize deep decarbonization in chemical manufacturing processes. Their approach combines new photocatalyst technology with a unique reactor design to produce hydrogen, ammonia, methanol, and other chemicals using common low‐cost materials, resulting in reduced emissions and waste.

**Figure 1:**
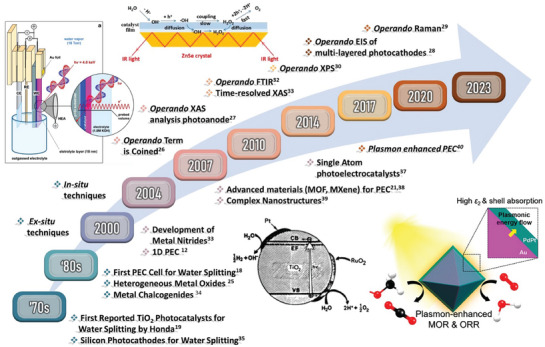
Timeline for materials development and Operando characterization milestones for PEC technologies. Bottom left schematic is Reproduced with permission.^[^ ^46^ ^]^ Copyright 1981, ACS Publications. Bottom right schematic is Reproduced with permission.^[^ ^47^ ^]^ Copyright 2023, ACS Publications. Top left schematic is Reproduced with permission.^[^ ^30^ ^]^ Copyright 2017, ACS Publications. Top right schematic is Reproduced with permission.^[^ ^48^ ^]^ Copyright 2021, ACS Publication.

Overall, advancements in PEC experienced a rapid evolution over the past few decades, leading to the development of diverse materials and paving the way for the widespread use of solar‐driven water‐splitting technology.^[^ ^12^ ^]^ Despite the advances made so far, PEC technologies still require breakthroughs in developing active materials and a better understanding of the underlying phenomena. A timeline of the major developments in the field is illustrated in **Figure** **1**.^[^ ^12^, ^18^, ^19^, ^20^, ^21^, ^22^, ^23^, ^24^, ^25^, ^26^, ^27^, ^28^, ^29^, ^30^, ^31^, ^32^, ^33^, ^34^, ^35^, ^36^, ^37^, ^38^, ^39^, ^40^ ^]^ It began in the early 1970s when Honda and Fujishima first investigated the potential of using titanium dioxide (TiO_2_) for solar‐driven water splitting.^[^ ^19^ ^]^ However, the efficiency of water splitting using metal oxide photocatalysts was low due to their large bandgaps and the relatively short lifetime of the charge carriers.^[^ ^41^ ^]^ In the 1980s and 1990s, researchers began to develop heterogeneous photocatalytic systems of metal oxides together with various materials that exhibited improved photochemical properties.^[^ ^20^, ^25^ ^]^ Silicon has also been explored as a promising PEC catalyst since the 1980s, offering potential advantages in terms of its abundance and favorable electronic properties.^[^ ^35^ ^]^ However, silicon cannot be used by itself as PEC due to low chemical stability but it is combined with other semiconductors and electrocatalysts.^[^ ^42^ ^]^ Later on, metal chalcogenides were discovered to possess superior photocatalytic properties compared to metal oxides.^[^ ^43^ ^]^ In the early 2000s, metal nitrides having a narrower bandgap compared to metal oxides were found to induce better light absorption and possess higher charge carrier mobilities, which led to enhanced photocatalytic performance.^[^ ^23^ ^]^ The first example of a non‐oxide photocatalyst based on RuO_2_ loaded β‐Ge_3_N_4_ was reported in 2005.^[^ ^44^ ^]^ Following that, a number of studies utilized group III‐V semiconductors as electrode materials for PEC and with the improvement of nanostructures, the performance of the aforementioned materials were enhanced to meet the photocatalytic requirements.^[^ ^45^ ^]^

Addition to advancements in materials development, *operando* characterization techniques began emerging as a powerful set of tools for understanding the behavior of PEC materials under working conditions. The term “*Operando*” was coined in 2001 to describe in situ measurements of catalysts during catalytic reactions.^[^ ^49^ ^]^ In the last decade, different *operando* techniques were applied for PEC for instance, including X‐ray Absorption Spectroscopy (XAS) for providing insights into the electronic and structural changes of photocatalysts,^[^ ^50^, ^51^, ^52^ ^]^ Infrared spectroscopy (FTIR) and Raman spectroscopy enabling the identification of reactive intermediates,^[^ ^29^, ^53^ ^]^ reaction pathways^[^ ^31^, ^54^ ^]^ and real‐time monitoring of the photocatalyst's surface chemistry.^[^ ^55^, ^56^ ^]^ X‐ray Photoelectron Spectroscopy (XPS) provides a deeper understanding of the surface chemical states and reaction mechanisms.^[^ ^30^ ^]^ Electrochemical Impedance Spectroscopy (EIS) provides valuable information on the charge and mass transport of the charge carriers.^[^ ^57^, ^58^ ^]^ *Operando* techniques so far have greatly contributed to our understanding of PEC processes and can aid in the development of more efficient and sustainable energy conversion technologies. However, the current state of the PEC technologies is still far from achieving the targets due to insufficient solar energy conversion efficiency, stability, and an adverse relation between the cost and durability of the semiconductor materials used in forming the electrode structures.^[^ ^13^ ^]^

In this review, we highlight the recent advancements made in developing PEC materials and advancements in *operando* investigations focusing on spectroscopic techniques. We aim to help readers become acquainted with various categories of materials used in photoelectrochemical H₂ production, along with the key governing parameters. Additionally, we emphasize the significant advancements that can be achieved through the use of operando characterization techniques, which we consider essential for progress in this field. Furthermore, we discuss potential improvements in both operando characterization methods and material development, highlighting areas where further advancements can be made to enhance the overall efficiency of photoelectrochemical H₂ production. We first explore the working principles of PEC and indicate the applied strategies for enabling better efficiency and stability of the photocatalytic materials. We compile and compare the most recently employed materials and different electrode designs where we classify the materials into transition metal oxides, transition metal chalcogenides, group IV, and group III‐V semiconductors. Next, we provide a critical review of the *operando* characterization techniques and emphasize their contribution to understanding the different processes of PEC materials including the formation of intermediates, the evolution of structures, degradation pathways and charge transport properties.

### 1.1 Working Principles of PEC

Photocatalysis (PC) uses semiconductor materials that can be photo‐excited in the range of near‐UV to near‐IR to produce electrons (e^‐^) and holes (h^+^) employed in tailored reactions. PEC is a method that improves regular PC electrochemically by applying an external potential bias to the photoelectrodes under light absorption.^[^ ^59^ ^]^ This helps increase the efficiency of the photocatalysis process by reducing the recombination of photogenerated (e^‐^/h^+^) pairs. Based on the literature, a maximum solar‐to‐hydrogen (STH) conversion efficiency of 9.2% was reported for PC systems,^[^ ^60^ ^]^ while in theory, PEC systems can potentially deliver nearly 25% STH efficiency with the help of applied external potential bias,^[^ ^61^ ^]^ as this bias increases the transfer rate of the remaining charge carriers on the photoelectrode surface, therefore reducing their recombination rate and improving the overall performance.^[^ ^62^ ^]^ Besides these theoretical projections, experimental STH efficiencies up to 13.5% have been reported for PEC systems in the literature, proving that PEC is more efficient than PC.^[^ ^63^, ^64^, ^65^ ^]^

**Figure 2:**
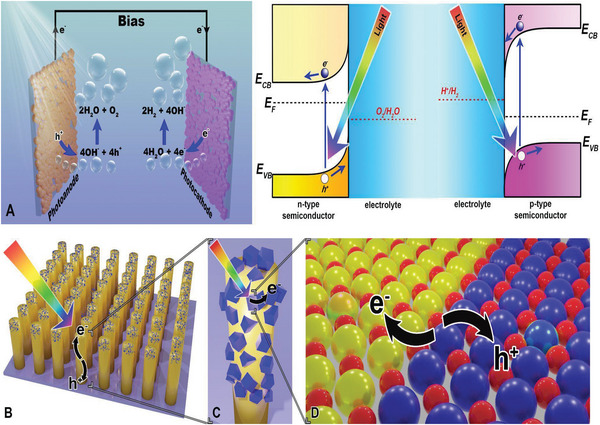
Mechanisms occurring in a PEC device. A) OER and HER reactions in the photoelectrochemical (PEC) system. Left panel: The scheme illustrates the main reactions happening in PEC water splitting, where photoanode and photocathode are in an aqueous electrolyte. First, light interacts with semiconductor materials, generating electron–hole pairs. On the photoanode while holes go to the surface, electrons go into the bulk and reach over to the counter electrode. On the photocathode, the reverse process occurs where holes on the surface react with OH^‐^ to generate O_2_ and electrons yield H_2_. Right panel: The band structure of the photoanode and photocathode are illustrated. With a n‐type semiconductor, after contact with the electrolyte, the electronic chemical potentials become equal, and the bands shift upward at the interface. This process facilitates electron movement into the bulk and hole movement toward the surface. For the photocathode where a p‐type semiconductor is used, the opposite process occurs. There are various ways to enhance the overall performance of the photoelectrodes as illustrated in (B)‐(D): First in B), Nanostructuring of the surface increases the contact area, and would also delay electron/hole recombination by reducing the charge carrier migration distance. And in faceted nanosized semiconductors, the charge carriers tend to move to different facets. In C), covering the surface with cocatalysts and photosensitizers would prevent electron‐hole recombination and broaden the light absorption spectrum. Finally, in D), creating a heterojunction between the semiconductors would enhance charge separation via band bending. Similarly, doping the semiconductor can increase the light absorption range and enhance the generation of electrons and holes.

**Figure** **2**a illustrates the components of a conventional PEC device which includes i) a semiconductor‐attached photoanode, where the hydrogen evolution reaction (HER) occurs; ii) a photocathode, where the oxygen evolution reaction (OER) occurs; and iii) the aqueous electrolyte. The spontaneity of water splitting is determined by the position of the conduction band (CB), the valence band (VB), and the stability of the semiconductor material in the PEC cell. Materials with corresponding band edges that take place between redox potentials for OER (1.23 V V_RHE_) and HER (0 V_RHE_) can be utilized in water splitting.^[^ ^59^ ^]^ The PEC process can be described in several steps: First, photons are absorbed by the semiconductor material which should possess higher energy than the bandgap energy of the semiconductor material (hv > E_bg_). This excites the VB electrons to the CB leaving holes behind, upon which the charge carriers can migrate to the surface and initiate electrochemical reactions, as illustrated in Figure 2a. These reaction steps are rather complex and occur within various time scales.^[^ ^66^ ^]^ When the photoanode is in contact with the electrolyte solution, there forms a Schottky junction at their interface, causing a change in the electrochemical potential (Fermi level) of the semiconductor when it is in equilibrium with the electrolyte. Then, “band bending” occurs, preventing the recombination of electron/hole pairs, where bending takes place in the space‐charge layer (SCL) region of the semiconductor.^[^ ^59^, ^62^ ^]^

The use of n‐type semiconductor leads to a lower Fermi level than the CB, causing oxidation to occur in the holes in VB, meanwhile, the electrons in the CB are collected by the external circuit, forming SCL in the semiconductor. An external potential bias can be applied to control the Fermi level of the semiconductor, thus controlling the band bending and the extent of the SCL. If E > E_fb_, electrons will be depleted, and holes will be enriched in the semiconductor.^[^ ^62^ ^]^ If E = E_fb_ with the same kind of semiconductor, then the electron bands will be flat, leading to recombination and annihilation of the charge carriers.^[^ ^59^ ^]^ The use of p‐type semiconductor leads to inverse processes that of n‐type. The difference between the processes utilizing the n‐type and p‐type semiconductors is demonstrated in the right panel of Figure 2a.

### 1.2 Challenges in PEC Materials Design

Four main properties determine the viability of semiconductor materials for PEC H_2_ generation. First, the semiconductor material should absorb a wide range of light which is directly related to the bandgap of the material. Second, the photoinduced (e^‐^/h^+^) pairs should exist for a sufficient length of time to be used for the intended reactions. This means the semiconductor material should provide a highly effective charge generation and transport features from the internal electrode to the photoelectric surface. Third, HER and OER reactions on the surface must be kinetically fast to allow for the efficient H_2_ generation process. Fourth, the material should have a sufficient operational life; long‐term stability and resistance to corrosion in acidic or basic environments is crucial for the material to operate effectively.^[^ ^67^, ^68^, ^69^, ^70^ ^]^

The ideal semiconductor material to be used for PEC hydrogen generation must have a bandgap range of 1.5 to 2.4 eV to be able to absorb a wide spectrum of light from solar radiation.^[^ ^71^, ^72^ ^]^ The lifetime scale of the photoinduced (e‐/h+) pairs should be long enough for them to be transported to the surface and injected into the electrolyte. The kinetics of HER and OER reactions are known to be sluggish due to the involvement of several mechanistic steps which require the use of a noble metal catalyst. Lowering the cost of materials to be used for PEC could indicate an advantage, however, useful lifetime and durability must exceed a certain threshold to render the technology competitive against other technologies available.^[^ ^73^, ^74^, ^75^ ^]^

PEC electrodes that possess a wide wavelength range of light absorption, induce efficient (e^‐^/h^+^) pairs generation, and enable fast surface reactions cannot be established in one semiconductor material. Designing viable materials that possess all of the aforementioned properties is a major challenge that hampers the development of PEC technologies for widespread adoption.^[^ ^76^, ^77^ ^]^ Existing semiconductor materials face several issues during operation which rule them out from being practical options, based on the intrinsic properties of the semiconductor materials used: fast (e^‐^/h^+^) pair recombination, slow hole injection into the electrolyte, the narrow wavelength range of light absorption due to the position of the bandgap edges, and poor chemical stability.^[^ ^73^, ^78^, ^79^ ^]^ For instance, TiO_2_ which is the first semiconductor material to be used for water splitting upon light irradiation has a narrow wavelength range of light absorption; TiO_2_ only absorbs the UV part of the electromagnetic spectrum and chemically degrades under acidic environments.^[^ ^80^ ^]^ Hematite (α‐Fe_2_O_3_) on the other hand absorbs in the visible region but suffers from very rapid charge recombination under illumination.^[^ ^81^ ^]^ Other materials such as non‐oxide semiconductors (Si, GaAs, InP, etc.) show better PEC properties but are unstable in acidic environments under illumination.^[^ ^82^ ^]^ The stringent requirements for economical and technologically effective PEC cannot be met with only one semiconductor material and needs improvement strategies and the use of several materials simultaneously in the same PEC electrode.

### 1.3 Materials Development

**Figure 3:**
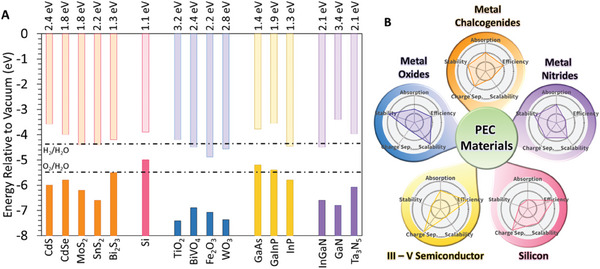
Comparison of the PEC materials. A) Bandgap values for different PEC material systems and the relative positions of the HER and OER reactions, B) A qualitative comparison of PEC material parameters.

The development of high‐performance PEC electrode materials is limited to semiconductor materials that can be excited by light illumination, including transition metal oxides, nitrides, chalcogenides, group IV and group III‐V compound semiconductors. bandgap energies of these materials are summarized in **Figure** **3**a which shows the valence and conduction band levels of different semiconducting materials with the thermodynamic levels of HER and OER reactions. As mentioned previously, a high‐performance PEC material must enable a wide range wavelength of light absorption to provide a high rate of electron‐hole pair formation with a slow recombination rate and fast charge injection. Meeting all these conditions in one material is a major challenge, if not impossible to achieve. To match these requirements, researchers have implemented different strategies in designing PEC devices with acceptable efficiencies and stabilities. The aim is to meet the targeted efficiencies with enough lifetime of operation to be economically competitive.

### 1.4 Performance Metrics

PEC electrode materials are assessed by metrics that provide information about their efficiency and stability. Photocurrent density and potential are the two main electrochemical parameters used to assess PEC materials. Photocurrent density is the amount of current per unit area generated upon irradiation of the electrode. In PEC the irradiation is accompanied by applying voltage bias. For that, applied bias photon‐to‐current efficiency (ABPE) is used which provides information about the amount of current density achieved along a voltage range normalized to the irradiation power.^[^ ^83^ ^]^ Incident photon‐to‐current efficiency (IPCE) is also used which is a measure of the photogenerated current for a given number of incident photons at a given wavelength.^[^ ^84^ ^]^ A key metric that is used to assess the applicability of a PEC material for H_2_ generation is solar‐to‐hydrogen (STH) efficiency. It is defined as the ratio of the produced chemical energy divided by the input solar energy. It is calculated by multiplying the system Faradaic efficiency by the ratio of electric power to light power. Faradaic efficiency is defined as the amount of hydrogen produced per measured current. This requires a measurement of the amount of H_2_ produced during irradiation.^[^ ^85^ ^]^ In PEC, STH should be measured at short circuit which further complicates the measurement. Alley et al. and previously Hu et al. reviewed the best practices used to assess the STH efficiency metric for PEC devices and provided protocols which facilitates benchmarking and the comparison of the performance of various PEC materials.^[^ ^84^, ^86^ ^]^

Potential values are used to assess the thermodynamic behavior of the PEC materials. Onset potential is used to determine the potential value at which the OER or the HER reactions start to produce photocurrent upon irradiation. Overpotential provides information about the amount of extra potential required to be applied due to the kinetic, conductivity or mass transfer limitations found in the system.^[^ ^67^ ^]^

The durability of the material is assessed by its chemical stability during operation. PEC materials face photo corrosion due to the self‐oxidation by the photo‐produced charges and the chemical/electrochemical side‐reactions with acidic/alkaline electrolytes.^[^ ^74^ ^]^ Stability is assessed by the lifetime of the materials to provide constant value of the photocurrent density under irradiation. Life‐time assessments are made by operating the PEC electrodes under illumination and applied bias in two or three electrodes set‐up.^[^ ^87^ ^]^ Then operation time is reported along the photocurrent density produced.

### 1.5 Performance Improvement Strategies

Theoretically a semiconducting material with a bandgap of 2.0 eV can achieve an STH conversion efficiency of ≈17.9% under 1.5 G illumination (100 mW cm^−2^).^[^ ^88^ ^]^ In practice, this is not achieved, and fewer STH percentages are obtained due to the issues mentioned in the challenges section. The strategies used to achieve higher STH efficiencies and to increase the material stability include nanostructure utilization, bandgap engineering, forming heterojunctions of different semiconducting materials, defect control, doping, catalyst loading and surface treatment of the electrode materials which will be mentioned briefly in the following paragraphs.

The aim of every strategy is to either overcome a drawback in a material or to enhance a certain property of another. Hybridization of different materials has served as an excellent strategy for that purpose. Forming a heterojunction by hybridizing different semiconductor materials allows for better charge transfer properties. It also contributes to the optimization of light absorption by allowing the extension of wavelength range. Moreover, forming heterojunctions results in the extension of the charge carrier's lifetime by directing the formed charges to certain junction.^[^ ^89^, ^90^, ^91^, ^92^ ^]^

Another approach is to adjust the surface of the semiconducting material for the aim of increasing stability and efficiency. The use of catalysts to enhance the kinetics of OER and HER reactions at the surface of the semiconducting material have shown increased performance. Noble metal catalysts such as Pt and Ru have been utilized targeting the sluggish kinetics of OER and HER reactions resulting in competing photocurrent densities.^[^ ^64^, ^93^, ^94^ ^]^ The stability of the semiconducting materials is improved by coating and passivating the surface with protective layers which prevents the self‐corrosion of the photoactive material by the photoproduced charge carriers. The protective layer is important for some semiconducting materials to sustain the acidic and alkaline environments.^[^ ^95^, ^96^, ^97^ ^]^ Furthermore, engineering the surface by means of nanostructures is followed in some studies to increase the photocurrent density and to direct the charge carriers toward a certain surface. For that tailored morphology nanoparticles with specific facets are utilized in the photoactive materials.^[^ ^98^, ^99^, ^100^, ^101^, ^102^, ^103^, ^104^, ^105^, ^106^, ^107^ ^]^

### 1.6 Light Harvasting Strategies

Optimizing light harvesting is crucial in the PEC process. Techniques such as applying anti‐reflective coatings, using photonic crystals, and incorporating plasmonics have demonstrated improvements in overall performance.^[^ ^108^, ^109^, ^110^, ^111^, ^112^, ^113^ ^]^ Anti‐reflective coatings reduce reflection and enhance light transmission into the active layer. Materials like silicon nitride (Si_3_N_4_), titanium dioxide (TiO_2_), and magnesium fluoride (MgF_2_) have been shown to boost light harvesting when applied. Multilayer or gradient index coatings can be engineered to span a wide range of wavelengths, thereby reducing reflection across the entire solar spectrum. Photonic crystals and plasmonic nanoparticles help concentrate and direct light within the photoelectrode. Photonic crystals can be designed to reflect specific wavelengths back into the photoelectrode, effectively increasing absorption. Incorporating metallic nanoparticles such as gold or silver on the surface or within the photoelectrode can induce localized surface plasmon resonances, which enhance the local electromagnetic field, thereby increasing light absorption near the nanoparticles and improving charge carrier generation.^[^ ^114^, ^115^, ^116^, ^117^ ^]^

Even with the application of such strategies, there is still a long path of improvements in the efficiency and stability of the semiconducting materials needed which require the utilization of advanced materials and novel strategies. In the following section, we will examine the recent developments made in applying several new strategies for different semiconductor materials, including transition metal oxides, nitrides, transition metal chalcogenides, Si, and group III‐V compound semiconductors. Figure 3b provides a spider chart comparing stability, efficiency, solar light absorption, charge separation, and scalability.

### 1.7 Transition Metal Oxides

Metal oxides have been the most widely studied materials for PEC hydrogen generation via water splitting. From environmental and economic perspectives, the non‐toxicity and natural abundance of these materials along with low cost make them favorable candidates both for lab‐scale PEC development and for large‐scale industrial applications. Rendering high photostability against UV and visible‐light illumination and better chemical stability compared to other photocatalysts, these materials are highly durable options with high catalytic activity.^[^ ^73^, ^118^ ^]^ N‐type metal oxide semiconductors, including TiO_2_, BiVO_4_, Fe_2_O_3,_ and WO_3_, have been commonly used as promising photoelectrodes for PEC water‐splitting applications.^[^ ^119^, ^120^, ^121^, ^122^ ^]^ TiO_2_ has gained particular attention as a low‐cost, versatile and chemically‐stable option with suitable band edge positions for water splitting.^[^ ^123^ ^]^ However, a major drawback with TiO_2_ is that its large bandgap of 3.0–3.2 eV limits light absorption to the UV range, and suffers from rapid recombination of photogenerated (e^‐^/h^+^) pairs.^[^ ^124^, ^125^ ^]^

**Figure 4:**
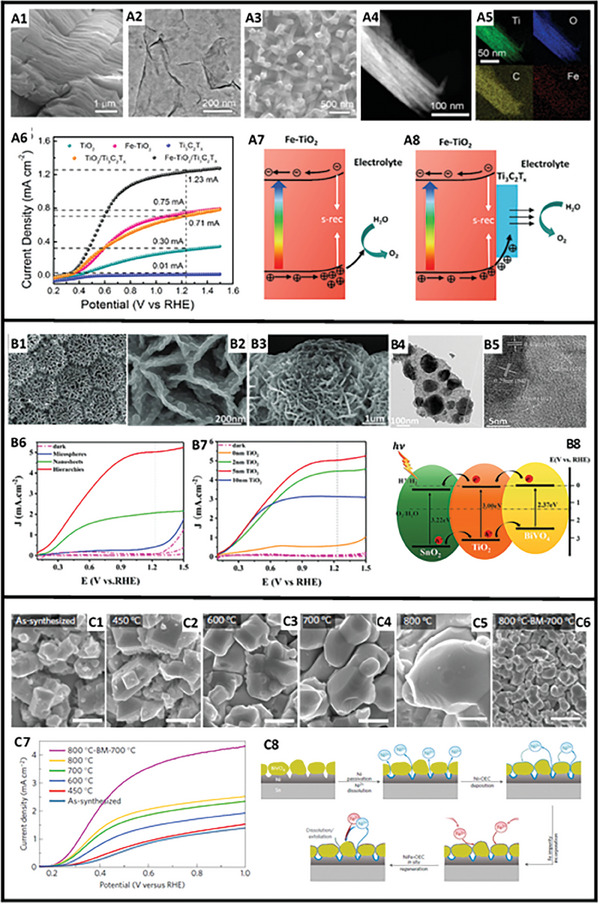
Examples of improvement strategies applied for enhancing the performance of transition metal oxide electrodes. A1) SEM image of as obtained Ti_3_C_2_. A2) TEM image of the exfoliated Ti_3_C_2_T_x_ nanosheets. A3) SEM image of the Fe‐TiO_2_/Ti_3_C_2_T_x_ structure. A4) HAADF‐STEM image of a single Fe‐TiO_2_/Ti_3_C_2_T_x_ structure. A5) Elemental maps of the Fe‐TiO_2_/Ti_3_C_2_T_x_ structure shown in (A4). A6) Light absorption and PEC performances of the as prepared TiO_2_, Ti_3_C_2_T_x_, TiO_2_/Ti_3_C_2_T_x_, Fe‐TiO_2_ (Fe: 5 mol.%), and Fe‐TiO_2_/Ti_3_C_2_T_x_ photoanodes in 0.1 m NaOH: Photocurrent density versus bias voltage under AM 1.5G illumination. Schematics of the photocarrier generation and transportation processes on A7) Fe‐TiO_2_ and A8) Fe‐TiO_2_/Ti_3_C_2_T_x_ photoanodes. (Reproduced with permission.^[^ ^130^ ^]^ Copyright 2022, Elsevier); B1–B3) SEM images of SnO_2_/TiO_2_/BiVO_4_ H‐NSs@HMs arrays: (B1, B2) top view and (B3) cross section view. B4,B5) TEM image and high‐resolution TEM images of SnO_2_/TiO_2_/BiVO_4_ H‐NSs@HMs arrays. B6,B7) LSV curves measured under AM1.5 simulated light illumination (100 mW cm^−2^) and dark conditions in 0.5 m Na_2_SO_4_ solution with 0.1 m Na_2_SO_3_ (B6) SnO_2_/TiO_2_/BiVO_4_ HMs, SnO_2_/TiO_2_/BiVO_4_ NSs, and SnO2/TiO2/BiVO4 H‐NSs@HMs arrays, (B7) Comparison of SnO_2_/TiO_2_/BiVO_4_ H‐NSs@HMs arrays with different TiO_2_ layer thickness. B8) The electronic transfer mechanism of SnO_2_/TiO_2_/BiVO_4_ H‐NSs@HMs arrays. (Reproduced with permission.^[^ ^89^ ^]^ Copyright 2020, Wiley). C1–C6) SEM images of bare 0.3 at. %Mo‐doped BiVO_4_ electrodes. Scale bars: 1 µm. C7) Current–potential curves for sulfite oxidation in 0.2 m sodium sulfite containing 1 m potassium borate buffer at pH 9 under AM 1.5G irradiation. C8) Schematic illustration of NiFe‐OEC self‐generation and in situ regeneration. (Reproduced with permission.^[^ ^99^ ^]^ Copyright 2016, Nature).

Different strategies have been proposed both to increase the absorption of solar radiation for PEC, and to promote the separation of (e^‐^/h^+^) pairs (please see the cited examples in **Table** **1**). A most commonly employed method is based on surface modification of TiO_2_ by using different types of light absorbers including narrow‐bandgap semiconductors.^[^ ^126^ ^]^ In addition, TiO_2_ can be doped with narrow‐bandgap materials as an efficient strategy for narrowing its bandgap, which improves the photocatalytic response by shifting light absorption into the visible region.^[^ ^127^, ^128^, ^129^ ^]^ Novel materials developed in the last decade including MXene and MOF have also been considered as popular choices to improve TiO_2_ performance. Yin et al. developed a Fe‐TiO_2_/Ti_3_C_2_T_x_ photoanode by utilizing both doping and surface modification strategies to extend the light absorption capability of TiO_2_ and to enhance the OER kinetics by increasing the photocarrier separation efficiency^[^ ^130^ ^]^ as shown in **Figure** **4**(A1–A8). A significant increase from 21.8% to 92.8% in the maximum IPCE values of TiO_2_ and Fe‐TiO_2_/Ti_3_C_2_T_x_ photoanodes was reported at 380 nm, along with a photoelectrochemical current density of 1.23 mA cm^−2^ at 1.23 V_RHE_ for the hybrid Fe‐TiO_2_/Ti_3_C_2_T_x_ Figure 4(A6). Another study reported a dramatically increased PEC activity with enhanced charge injection and separation efficiency achieved through the synergistic effects between Ni and Fe active sites in octahedrally coordinated ultrathin NiFe‐MOF decorated TiO_2_ nanorods.^[^ ^131^ ^]^ NiFe‐MOF/TiO_2_ hybrid photoanode exhibited 3.35 times higher photocurrent density of 0.77 mA cm^−2^ at 1.23 V_RHE_ and a decreased onset potential with a cathodic shift from 0.36 to 0.29 V when compared to bare TiO_2_, indicating a facilitated PEC water oxidation.

**Table 1:** Comparison of recent advanced PEC electrode materials (*Light source: AM 1.5G front illumination unless otherwise stated) (J_ph_: photocurrent density, V_on_: onset potential, ƞ_sep_: charge separation efficiency, Applied bias photon‐to‐current efficiency (ABPE), Incident photon‐to‐current efficiency (IPCE)).

Material type	PEC catalyst	Improvement strategy	Bandgap [eV]	Electrolyte	J_ph_[mA/cm^2^] (at 1.23 V_RHE_)	V_on_ (V_RHE_)	Efficiency			Stability [h]	References
							ABPE	IPCE (at 1.23 V_RHE_)	ƞ_sep_ (at 1.23 V_RHE_)		
Metal Oxides	BiVO_4_–N,S co‐doped carbon nanosheet /PANI@CoPi	Light harvesting capability and performance improvement by anchoring N,S co‐doped carbon nanosheet to BiVO_4_	2.4	0.1 m potassium phosphate 0.1 m potassium phosphate with H_2_O_2_	4.46 ≈ 5.2	0.30	1.13% at 0.74 V_RHE_	71.32% (350 nm)	ƞ_surface_: 82.1% η_bulk_: ≈80%	10	[138]
	Co‐Pi/Ti_3_C_2_T_X_/α‐Fe_2_O_3_	Improved hole mobility and charge transfer efficiency achieved by inserting MXene nanosheets as mediators between α‐Fe_2_O_3_ nanorods and Co‐Pi layer	2.09	1 m NaOH	3.20	0.55	0.49% at 0.93 V_RHE_	56.1% (360 nm)	44%	20	[38]
	Fe‐TiO_2_/Ti_3_C_2_T_x_	Accelerated OER kinetics and increased photocarrier separation through the electrophoretic deposition of Ti_3_C_2_T_x_ on Fe‐TiO_2_	3.01	0.1 m NaOH 0.5 m Na_2_SO_3_	1.23 ≈ 1.40	–	0.50% at 0.68 V_RHE_	92.8% (380 nm)	87.4%	5	[130]
	NiFe‐MOF/TiO_2_	Improved PEC activity by introduction of abundant active sites on the photoanode surface through NiFe‐MOF loading on TiO_2_	2.98	0.5 m Na_2_SO_4_ 0.5 m Na_2_SO_4_ with H_2_O_2_	0.77	0.25	–	42.1% (390 nm)	37.4%	4	[131]
	SnO_2_/TiO_2_/BiVO_4_ Hierarchical Nanosheets@Hollow microspheres	Improved charge transport efficiency, light harvesting efficiency, and charge separation efficiency by 3D hierarchical ternary SnO_2_/TiO_2_/BiVO_4_ photoanodes	2.4	0.5 m Na_2_SO_4_ 0.1 m Na_2_SO_3_	3.10	–	–	85% (350 nm)	84.9%	5	[89]
	F:FeOOH/BiVO_4_/WO_3_	Doping fluorine into FeOOH for boosting OER activity	2.69 (WO_3_) 2.4 (BiVO_4_)	0.1 m potassium phosphate buffer	3.1	≈0.55	0.57% at 0.89 V_RHE_	36% (450 nm)	66.8%	3	[98]
	Mo:BiVO_4_/Ni/Sn	Improving the stability of the BiVO_4_ via a high‐temperature treatment and in situ catalyst regeneration by the use of Ni as contact layer	2.4	1 m potassium borate buffer	≈4.4	≈0.25	1.6% at 0.60 V_RHE_	73% (380 nm)	–	1100	[99]
	NiFe(OH)x/Ta:Fe_2_O_3_@Fe_2_O_3_	Enhancing the photocurrent density and reducing the turn‐on voltage via gradient doping, homojunction formation, and cocatalyst modification	2.1	1 m KOH	3.22	0.55	0.55% at 0.90 V_RHE_	≈32% (340 nm)	ƞ_surface_: 88.7% at 1.20 VRHE ƞ_bulk_: ≈37% at 1.25 VRHE	5	[141]
	BiVO_4_/poly(3‐hexylthiophene)‐CuPc/NiCo‐layered double hydroxide	The use of p‐type poly(3‐hexylthiophene) as hole transport layer to improve the surface OER kinetics and the suppression of charge recombination of the photoanode	2.55	0.5 m potassium borate buffer 0.5 m Na_2_SO_3_	4.25 5.54	0.23	1.36% at 0.65 V_RHE_	≈60% (≈460 nm)	73.8%	8	[139]
Nitrides	Mg:Ta_3_N_5_	Suppressing the defect‐related charge recombination via gradient Mg doping to increase the photocurrent density	2.1	1 m KOH	8.5	0.4	3.31% at 0.74 V_RHE_	≈82% (≈450 nm)	–	5 (80% retention after 13.5 h)	[100]
	In:GaN/Ta_3_N_5_/Mg:GaN	Forming heterojunctions through interface engineering to enhance charge separation efficiency and for a better surface charge injection efficiency	In:GaN: 3.3 Mg:GaN: 3.3 Ta_3_N_5_: 2.1	1 m KOH	9.3	0.38	3.46% at 0.74 V_RHE_	≈90% (≈420 nm)	≈75%	≈2.7 (80% retention after 10 h)	[90]
	BiVO_4_/BNNPs/CoCr‐LDH	Applying thin layer of 2D hexagonal boron nitride nanoplatelets for the surface modification of BiVO_4_ photoanodes to improve the photocvurrent density	2.4‐2.6	0.1 m Na_2_SO_4_	3.8	≈0.39	–	69% (350 nm)	64%	3	[93]
Metal Chalcogenides	In_2_S_3_/ZnIn_2_S_4_	Heterojunction and directed growth of In_2_S_3_ nanosheet arrays	2.09	0.1 m Na_2_SO_4_	1.22	–	0.56% at 0.39 V_RHE_	Faraday efficiency of 93%	–	–	[95]
	Cd‐ZnIn_2.2_S_y_ Nanosheets	Vertical growth of Cd‐ZnIn_2.2_S_y_ nanosheets	–	0.5 m Na_2_SO_3_ and 3.5% NaCl	5.85	0.78	≈3.10% at 0.53 V_RHE_	–	–	–	[181]
	MoS_2_/CdSe/TiO_2_	MoS_2_/CdSe/TiO_2_ ternary composites structure	TiO_2_ (≈3.2 eV) CdSe (≈1.7 eV) MoS_2_ (≈1.9 eV)	0.5 m Na_2_SO_4_	3.62 for 5% MoS_2_/10% CdSe/TiO_2_	1.0	–	Faraday efficiency of 90.8% (2 h)	–	–	[162]
	CdIn_2_S_4_	Ternary metal sulfide (CdIn_2_S_4_) with surface sulfur vacancies	1.97	0.5 m Na_2_SO_4_	5.73	1.23	2.49% at 0.477 V_RHE_	–	–	15 h	[165]
	MoS_2_/GaN	MoS_2_/GaN heterostructure	1.88	1 m NaOH	5.2	0.0	0.91% 0.21 V_RHE_	–	–	–	[91]
Silicon	Pt deposited, double‐sided Si	Chemically passivating both sides of Si to induce band offset for the separation of photogenerated carriers, furthermore, depositing ITO layers on both sides of Si as the carrier collector and anti‐reflection layer	1.1	1 m KOH	38.5	0.75	5.8% at 1.00 V_RHE_	–	–	200 h	[94]
	NiFe/Si	Depositing Ni/Fe catalyst onto Si wafers to improve the efficiency of the photoanode; as well as optimizing Ni:Fe ratio to get the best PEC performance	1.1	1 m KOH	29.5	0.96	3.12% at ≈1.1 V_RHE_	≈81% (800 nm)	–	14 h	[170]
	NiFeP/n‐Si	Reducing the recombination ratio of charges and the bandgap by implementing NiFeP nanoparticles on Si	0.24	1 m KOH	40	≈1.48	0.37% at 1.18 V_RHE_	87.8% (700 nm)	–	17 h	[96]
	Si w/GaN protective layer	Improving the charge carrier transfer process of Si by implementing a protective GaN layer	1.1	0.5 m H_2_SO_4_	38	0.5	10.5% at 0.32 V_RHE_	≈80% (≈620 nm)	–	113 h	[169]
	InGaN/GaN/Si	Improving charge transfer properties by exploiting the lateral carrier extraction scheme of 1D nanowire structures	InGaN: 2.39 Si: 1.1	1 m HBr	40.6	≈0.43	8.7% at 0.33 V_RHE_	72.3% (520 nm)	–	3 h	[171]
	p‐Si w/TiO_2_ protective layer	Depositing Pt and TiO_2_ to improve photoelectrochemical performance	1.1	1 m HClO_4_	31	0.48	5.9% at ≈0.27 V_RHE_	≈80% (600 nm)	–	90 h	[97]
III‐V Semiconductors	GaAs/IrO_2_	Using IrO_2_ as a co‐catalyst to improve the OER	≈1.42	0.5 m H_2_SO_4_	23.1	1.022	–	≈76% (700 nm)	–	192 h	[64]
	GaInP_2_/MoS_x_/TiO_x_	Deposition of MoSx films, then atomic layer deposition of TiO_2_ to improve the catalytic activity with the former, as well as the stability with the latter coating	GaInP2: ≈1.85 MoSx: 1.8 TiOx: 3.2	0.5 m H_2_SO_4_	11.2	0.4	–	≈78% (420 nm)	–	20 h	[174]
	InP	Utilization of InP nanopore arrays to benefit from their light‐trapping characteristics, as well as their conducting channels and large surface area	1.34	0.35 m Na_2_S + 0.5 m Na_2_SO_3_	27.5	0.45	≈6.7% at 0.35 V_RHE_	–	–	1.1 h	[101]
	InGaN/IrO_2_	Using IrO_2_ as a co‐catalyst to improve the OER	1.7	0.5 m H_2_SO_4_	10.9	0.1	3.6% at ≈0.73 V_RHE_	93% (440 nm)	–	10 h	[61]
	InGaP/GaAs	Decoupling the light harvesting component, thus improving the efficiency and stability, meanwhile reducing the cost	InGaP: 1.85 GaAs: 1.4	0.5 m KOH	9.8	≈0.5	–	InGaP: 68.5% (≈500 nm) GaAs: 79.9% (≈680 nm)	–	150 h	[173]
	InGaN/GaN	Using parallel illumination to improve the optimization of photovoltage and photocurrent	≈2.0	1 m HBr	≈43	0.7	≈2% at 0.6 V_RHE_	≈65% (≈350 nm)	–	–	[92]
	GaN protected GaInP_2_/GaAs/Ge	Implementing a GaN protective layer to protect the photoanode from photocorrosion and oxidation, as well as to prevent recombination of the charge carriers	GaInP2: ≈1.86 GaAs: ≈1.42 Ge: ≈0.65	0.1 m H_2_SO_4_	≈10.3	≈2.2	13.5% at ≈1.4 V_RHE_	–	–	80 h	[45]

Designing heterostructures for the development of 0D, 1D, 2D, and 3D nanostructured electrodes, which is generally combined with interface engineering, is another effective strategy to improve the PEC performance of metal oxide photoanodes. Cui et al. fabricated a 3D hierarchical ternary SnO_2_/TiO_2_/BiVO_4_ arrayed photoanodes for PEC water splitting^[^ ^89^ ^]^ where the hole blocker TiO_2_ is sandwiched between the conductive skeleton of hierarchical SnO_2_ hollow spheres forming nanosheet shell arrays and the photon absorber BiVO_4_. The improved light harvesting efficiency of SnO_2_/TiO_2_/BiVO_4_ was attributed to the highly porous large surface area of SnO_2_ and cascade structured band alignment of TiO_2_/BiVO_4_ and TiO_2_/SnO_2_, through which the charge carrier separation process was significantly improved and the (e^‐^/h^+^) pairs recombination rate was suppressed shown in Figure 4(B1–B8).^[^ ^89^ ^]^

Naturally‐abundant BiVO_4_ has shown the most promising PEC performance so far among the metal oxides due to its appropriate bandgap of 2.4 eV for the adsorption of visible light up to 520 nm and reasonable band‐edge positions with respect to the water oxidation and reduction potentials.^[^ ^132^ ^]^ However, its moderate hole diffusion length (≈70–100 nm), low electron mobility (≈10^‐2^ cm^2^ V.s^−1^), sluggish charge transfer due to a polaron hopping instead of a band‐conduction, and poor OER kinetics have been limiting its performance.^[^ ^133^, ^134^ ^]^ Another major drawback of BiVO_4_ is the V^5+^ ion dissolution, photocorrosion therefore remains to be a major challenge.^[^ ^135^, ^136^ ^]^ BiVO_4_’s PEC performance has been improved through utilizing the combination of different strategies including the use of a suitable OER co‐catalyst, such as a cobalt (oxy)hydroxide phosphate water oxidation electrocatalyst (CoPi) for surface modification.^[^ ^137^ ^]^ Reddy et al. improved the hole transfer efficiency by connecting BiVO_4_ and CoPi with flexible polyaniline (PANI) which acts both as a protection layer for BiVO_4_ and provides 10 hours of stability, and as a hole transfer layer between BiVO_4_ and CoPi, increasing the photocurrent density up to 4.46 mA cm^−2^ at 1.23 V_RHE_ in a 0.1 m potassium phosphate electrolyte.^[^ ^138^ ^]^ The authors also anchored N and S co‐doped carbon nanosheets (NSCN) to BiVO_4_ as a non‐toxic visible‐light absorber. The resulting BiVO_4_−NSCN/PANI@CoPi achieved an onset potential of 0.30 V, and IPCE value of 71.32% at 350 nm and ABPE of 1.13% at 0.74 V_RHE_.^[^ ^138^ ^]^ Layered‐double hydroxides (LDH) such as NiCo‐LDH, CoFe‐LDH, NiFe‐LDH, MnNi‐LDH are also suitable as OER co‐catalysts to promote water oxidation on the surface of BiVO_4_‐based photoelectrodes. Further improvements are underway to overcome the sluggish OER kinetics of BiVO_4_‐based photoelectrodes; for example, Pei et al. developed an intrinsic p‐type polymer (poly(3‐hexylthiophene) (P3HT) doped with tetra‐tert‐butyl substituted copper (II) phthalocyanine (CuPc) as a hole transport layer for a BiVO_4_/NiCo‐LDH composite photoanode to promote the photoinduced holes to efficiently participate in the OER.^[^ ^139^ ^]^

Forming heterojunctions is another efficient strategy to improve electron‐hole charge separation kinetics and the overall PEC efficiency. One of the most promising heterojunctions is the WO_3_/BiVO_4_ system given the well‐matched positions of their conduction band (CB) and valence band (VB) edges. Li et al. prepared such a composite photoanode by forming a BiVO_4_/WO_3_ heterojunction followed by incorporating a F‐doped FeOOH co‐catalyst to form a composite photoanode of F:FeOOH/BiVO_4_/WO_3_.^[^ ^98^ ^]^ They ascribed the reported photocurrent density of 3.1 mA cm^−2^ as 7 times and 9 times that of bare WO_3_ and BiVO_4_, respectively, to the hole extraction capability of F:FeOOH.^[^ ^98^ ^]^ Stability of the photoelectrodes is another important issue that needs to be solved for the practical and long‐term use of PECs.^[^ ^99^ ^]^ As simulated using a Pourbaix diagram, BiVO_4_ is thermodynamically stable at low values of external voltages and in near‐neutral or neutral electrolytes, and it begins to decompose in acidic or basic environments and at potentials levels close to that of oxygen evolution.^[^ ^140^ ^]^ To overcome the stability issues of the photoelectrodes, researchers have been focusing on developing metal oxide‐based photoelectrodes with thermodynamic stability over a broad range of pH or potential levels, and promising results have been reported. Kuang et al. synthesized a highly crystalline Mo‐doped BiVO_4_ photoelectrode, shown in Figure 4(C1–C6), via a two‐step annealing process. First, they synthesized Mo‐doped BiVO_4_ by reacting Mo‐doped K_3_V_5_O_14_ and Bi(NO_3_)_3_·5H_2_O. After annealing at 800 °C for 2 h, the as‐synthesized Mo‐doped BiVO_4_ particles were ball‐milled twice at 800 rpm for 30 min, and then re‐annealed at 700 °C for 2 h.^[^ ^99^ ^]^ Subsequently, they prepared a NiFe‐OEC/Mo:BiVO_4_/Ni/Sn electrode (OEC: oxygen evolution catalyst) by using Ni as the contact layer, and achieved an outstanding stability of 1,100 h with a photocurrent density of 2.6 mA cm^−2^ at 0.6 V_RHE_ in 1 m borate buffer under AM 1.5G irradiation through an in situ catalyst regeneration process Figure 4(C7). Comparing this performance level to the case where Ti is used as the contact layer, a considerable degradation occurs only after 10 h under similar testing conditions. This exceptional stability has been attributed to the catalyst regeneration processes of NiFe‐OEC/Mo:BiVO_4_/Ni/Sn catalyst as shown in Figure 4(C8), comprising four steps: i) Ni passivation and Ni^2+^ ion dissolution; ii) photo electrodeposition of Ni‐OEC; iii) Fe^2+^ incorporation from the electrolyte containing 10 µm of Fe^2+^ to form NiFe‐OEC and iv) self‐regeneration of NiFe‐OEC to prevent OEC loss during the long‐term operation.^[^ ^99^ ^]^

Hematite (α‐Fe_2_O_3_) exhibits a bandgap of 2.1–2.2 eV and is widely used in PEC applications given its several advantages being one of the most earth‐abundant materials and possessing high chemical stability under a range of operating conditions. Its theoretical solar‐to‐H_2_ efficiency has been reported to be ≈15%, however, experiments show that its poor optoelectronic properties, such as very short (up to 4 nm) diffusion length of holes and the position of its conduction band (+0.28 V_RHE_) result in a relatively high OER overpotential which limits the photocurrent output. Recent studies demonstrated that designing interfacial charge modulation systems using both conventional Co‐Pi OEC layers and novel materials such as MXene nanosheets (MNs) as hole mediators can help reducing the charge recombination rate by forming a MNs/α‐Fe_2_O_3_ Schottky junction and facilitate hole transfer from α‐Fe_2_O_3_ to the Co‐Pi surface. Consequently, the Co‐Pi/MNs/α‐Fe_2_O_3_ photoanode retained 90% of the initial photocurrent density after 20 h of consecutive irradiation, and possessed a photocurrent density of up to 3.20 mA cm^−2^ at 1.23 V_RHE_, almost 4 times that of bare α‐Fe_2_O_3_ (0.84 mA cm^−2^); an onset potential of 0.57 V_RHE_ (having exhibited a large cathodic shift of ≈250 mV); an ABPE of 0.49% at 0.93 V_RHE_; and an IPCE of 56.1% at 360 nm.^[^ ^38^ ^]^ Forming co‐catalyst modified gradient Ta‐doped homojunctions has also been reported to create an intrinsic built‐in electric field where the surface states are passivated to suppress charge carrier recombination in Fe_2_O_3_. The optimized photoanode of NiFe(OH)_x_/Ta:Fe_2_O_3_@Fe_2_O_3_ exhibited a considerably low onset potential of 0.55 V_RHE_ with a cathodic shift by ≈270 mV and a photocurrent density of up to 3.22 mA cm^−2^ at 1.23 V_RHE_.^[^ ^141^ ^]^

### 1.8 Transition Metal Chalcogenides

Transition metal chalcogenides are semiconducting materials that have chalcogens (S, Se, Te) as anions. They can be found in various minerals and stoichiometric forms with two and 3D crystalline structures. Their optoelectronic properties can be modulated by changing their chemical composition which can exist in the form of metal mono (M_X_), di (MX_2_), and tri (MX_3_) chalcogenides. Their bandgap energies lie between 0 and 2 eV with large values of the light absorption coefficient. The CB and the VB alignment is suitable for the water‐splitting reactions and their exposed surface atoms (S, Se, Te) represent highly active sites for water‐splitting reactions due to higher electronegativity, weaker hydrogen bonding, and higher percentage of exposed atoms.^[^ ^142^, ^143^, ^144^, ^145^ ^]^ Overall, these properties make metal chalcogenides promising photoanode materials for PEC. Examples of chalcogenides used in PEC applications include CdS, InSe, PbS, MoS_2_, SnS_2_, Bi_2_S_3_, Sb_2_Se_3_, Bi_2_S_3_, and In_2_S_3_.^[^ ^146^, ^147^, ^148^ ^]^ Earlier studies have shown that single‐structure transition metal chalcogenides such as MoS_2_ and WSe_2_ provide high power conversion efficiencies.^[^ ^149^ ^]^ Later studies have shown that adding a catalyst (Pt and Ru) to crystalline p‐type WSe_2_ can provide relatively high photocurrent densities.^[^ ^150^ ^]^ Recent work focused on taking advantage of the layered structure of metal chalcogenides via exfoliation and layer transfer to obtain multilayer nanostructured photoanodes.^[^ ^151^, ^152^, ^153^, ^154^, ^155^ ^]^

The main challenges for the utilization of transition metal chalcogenides in PEC electrodes are due to their poor long‐term stability, poor conductivity, and complicated synthesis. Metal sulfides in general are thermodynamically less stable compared to metal oxides under oxidizing potentials.^[^ ^156^ ^]^ Several studies suggested that chalcogenides undergo morphological evolution, reconfiguration of electronic properties such as density of states, and restructuring of surface dangling bonds during the operation of PEC electrodes.^[^ ^157^, ^158^ ^]^ Several other studies suggested that the overall stability could be related to the conversion of metal chalcogenides to more stable oxide forms during the early cycles of operation.^[^ ^159^, ^160^ ^]^ Therefore, researchers have developed material design methods that target the enhancement of chemical stability along with increasing efficiency. The main strategy followed for enhancing the performance is forming heterojunctions with different metal chalcogenides or with different semiconductors such as metal oxides and applying protective layers.^[^ ^95^, ^161^, ^162^, ^163^ ^]^ Several studies that demonstrate the enhancement of efficiency and stability of metal chalcogenides are summarized in Table 1. An example of heterostructure with a metal oxide is based on the study done by Liu et al, in which the authors synthesized 2D ZnIn_2_S_4_ nanosheets with 1D TiO_2_ nanorod heterostructure arrays that demonstrated enhanced photocurrent density and a negative shift in the onset potential.^[^ ^161^ ^]^ Another study by Wang et al included the synthesis of a ternary composite (MoS_2_/CdSe/TiO_2_) photoanode which resulted in increased light absorption and enhanced photocurrent density.^[^ ^162^ ^]^ Li et al. prepared a heterojunction of WO_3_ with CdS modified with dual co‐catalysts NiOOH and Co‐Pi and reported a substantially improved photocurrent density, ≈6.4 times compared to that of bare WO_3_.^[^ ^164^ ^]^

Recent examples of heterojunction structures of different metal chalcogenides include the preparation of epitaxial In_2_S_3_/ZnIn_2_S_4_ heterojunctions by Geng et al.^[^ ^95^ ^]^ The proposed structure showed a larger specific surface area and a stronger ohmic contact with the conductive substrate compared to the In_2_S_3_ and ZnIn_2_S_4_ structures resulting in faster kinetics and more exposure to active sites. This was reflected in the PEC performance via 1.2 times increase in photocurrent density. Another such example by Li et al. involves an engineered e interfacial band structure for ZnIn_2_S_4_/SnS_2_ which showed suppression of bulk recombination, increasing active sites and decreasing the overpotential.^[^ ^163^ ^]^ Their approach was based on the introduction of the oxygen element at the interface to achieve bilateral band regulation in a nanosheet morphology which contributed also to increasing the active sites by exposing the In─O─Sn bond at the interface. Hassan et al introduced a novel MoS_2_/GaN heterostructure that demonstrated higher performance than single structures.^[^ ^91^ ^]^ They deposited MoS_2_ on single‐crystal GaN which resulted in a 5.2 mA cm^−2^ photocurrent density that is higher than the current obtained from GaN electrodes. The enhanced performance was related to the enhanced light absorption by the MoS_2_ layer which has reduced the charge transfer resistance between the semiconductor and the electrolyte interface and the better charge separation due to the presence of the heterostructure. In another study, Wang et al synthesized a highly active sulfur‐deficient ternary sulfide photoanode based on CdIn_2_S_4_ for which surface sulfur vacancies had a positive effect on interfacial charge separation and transfer kinetics.^[^ ^165^ ^]^ The sulfur vacancies were attributed to the charge accumulation on the adjacent In and Cd atoms which resulted in the formation of active sites for the OER intermediates. The vacancies were also related to the increased charge separation and enhanced charge injection by forming shallow trap states on the surface. The photoelectrode tested for PEC provided a 5.73 mA cm^−2^ photocurrent density at 1.23 V_RHE_. Furthermore, a recent study by Ahmad et al. demonstrated the impact of CdS and CdSe quantum dots in improving the performance of g‐C3N4 nanosheets in PEC applications. The creation of type‐II and Z‐scheme heterojunction systems was found to enhance charge separation leading to improved H_2_ production.^[^ ^166^, ^167^ ^]^

### 1.9 Silicon

Silicon is widely used in photoelectrochemistry research due to its narrow bandgap of ≈1.1 eV, abundance on Earth, low cost, operation flexibility on the solar spectrum, suitable position of conduction band edge for hydrogen evolution reaction, and production maturity for the photovoltaic (PV) industry.^[^ ^78^, ^94^, ^168^ ^]^ While a Si photoelectrode is able to demonstrate excellent photoelectrochemical performance by maintaining over 40 mA cm^−2^ current density, it is prone to oxidation or chemical dissolution when in contact with the electrolyte, thus having a low chemo‐mechanical stability.^[^ ^78^, ^169^ ^]^ Using thin film protective layers has been adopted to mitigate this instability issue, however, the thickness of the layer must be tuned carefully not to reduce light absorption. Several studies have shown that, photoelectrochemical performance of Si photoelectrodes was negatively impacted because of an excessively thick protective layer resulting in a drop in applied bias photon‐to‐current efficiency and the level of photocurrent.^[^ ^78^, ^94^, ^169^ ^]^

**Figure 5:**
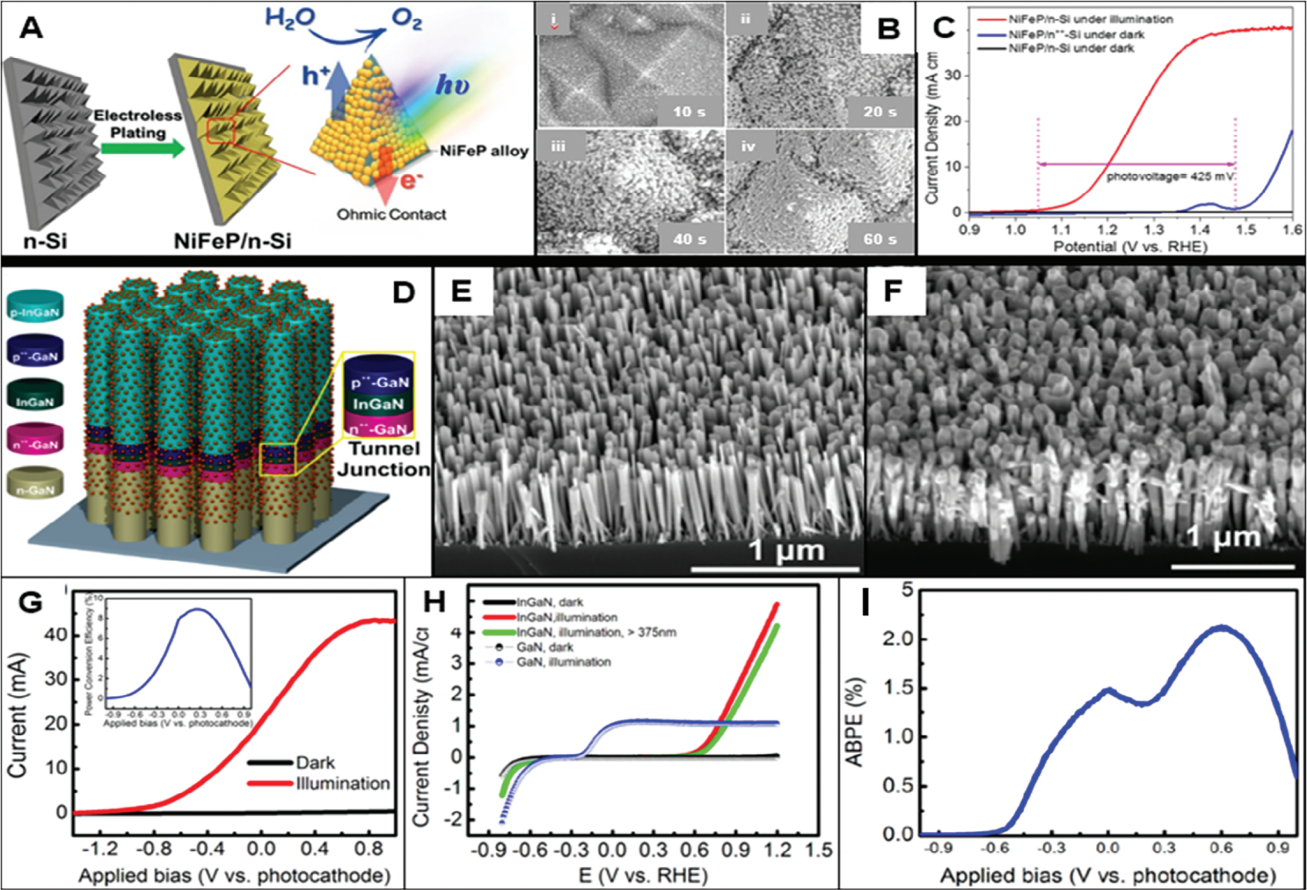
Examples of the applied improvement strategies for enhancing Si‐based and III‐V‐based photoelectrochemical system performance A) Process of the preparation of NiFeP/n‐Si photoanodes; B) FE‐SEM images of the NiFeP deposited silicon electrodes with deposition time of (a) 10 s, (b) 20 s, (c) 40 s, and (d) 60 s; C) polarization curves for NiFeP/n‐Si photoelectrode under simulated sunlight and the electrocatalytic activity of NiFeP/n^++^‐Si electrode in 1.0 m KOH. Reproduced with permission.^[^ ^96^ ^]^ Copyright 2020, ACS publishing. D) Schematic of the p‐InGaN nanowire photocathode connected directly with a low resistivity n‐Si substrate through a polarization‐enhanced tunnel junction and deposited Pt nanoparticles on the structure; E) SEM image of as‐grown GaN nanowires on n‐Si substrate with a tilt of 45°; F) SEM image of p‐InGaN nanowires grown on n‐Si substrate and deposited Pt nanoparticles with a tilt of 45°; G) *I*–*V* curve of the paired GaN nanowire photoanode and InGaN nanowire photocathode under dark and illuminated state; H) polarization curves of the GaN and InGaN nanowire photoanodes under dark and illuminated state; I) ABPE of the dual‐photoelectrode device. Reproduced with permission.^[^ ^92^ ^]^ Copyright 2015, ACS publishing.

Given their high conductivity, metals are commonly utilized as protective layers without sacrificing charge carrier transport properties of Si. Pt and Ni are commonly used to increase the rate of HER and to protect the Si photoelectrode from oxidation and rapid etching.^[^ ^78^, ^94^ ^]^ For example, Liu et al. chemically passivated both sides of the photoelectrodes to induce a band offset for the separation of photogenerated carriers. Furthermore, Pt coatings on Si photoelectrodes demonstrated high photocurrent density of 38.5 mA cm^−2^ and superior chemo‐mechanical stability of 200 h in an alkaline environment.^[^ ^94^ ^]^ When Ni‐based alloys are used as a catalyst and protective layer on Si, even though the resulting photoelectrode showed less stability compared to Pt‐coated electrodes, they provided high IPCE and photocurrent density; as demonstrated by Li et al. using NiFe, and Sun et al. using NiFeP, both in alkaline environments which is shown in **Figure** **5**A–C.^[^ ^96^, ^170^ ^]^

Apart from metals, semiconducting materials have also been proposed as used as protecting layers on Si photoelectrodes. Vanka et al. improved the charge carrier transfer process by implementing a protective GaN layer, where the electrode material exhibited 38.5 mA cm^−2^ photocurrent density, high IPCE, and a superior stability of 113 h in an acidic environment.^[^ ^169^ ^]^ In another work which utilized an InGaN/GaN/Si junction by Fan et al. a photocurrent density of 40.6 mA cm^−2^ was demonstrated in an acidic environment along with high ABPE and IPCE values as well exhibits as well, however with lower stability of 3 h.^[^ ^171^ ^]^ Gong et al. utilized a 26.4 nm thick TiO_2_ protective layer on their Si photocathode which exhibited high photocurrent density and IPCE, and stability of 90 h in an acidic environment.^[^ ^97^ ^]^ A comparison of selected parameters for the aforementioned studies is provided in Table 1.

### 1.10 III–V Semiconductors

Group III‐V semiconductors are regarded for their high efficiencies for both single‐junction and multiple‐junction photoelectrochemical cells. A photon‐to‐electron efficiency of 47.1% was demonstrated for a III‐V semiconductor‐based multiple‐junction photoelectrochemical cell, which is remarkably higher than the maximum 30% of Si‐based cells.^[^ ^45^, ^172^ ^]^ Tunability of their optoelectronic properties such as charge recombination coefficient, the ability of high light absorption, and exceptional charge transport properties render group III‐V semiconductors favorable in PEC research.^[^ ^64^, ^78^, ^173^ ^]^ However, III‐V semiconductors are in general very expensive, and rapidly deteriorate by etching when in direct contact with highly acidic or alkaline electrolytes, reducing the overall performance of the PEC system.^[^ ^45^, ^65^, ^78^, ^173^ ^]^

To alleviate the etching issues, semiconductor materials are often coated with a protective layer that is durable in harsh photocatalysis environments.^[^ ^65^ ^]^ Furthermore, catalytically active materials such as Pt, Ru, and Ir might be deposited on the surface of III‐V semiconductors to increase the rate of HER, but the use of those noble metals drives the manufacturing cost much higher.^[^ ^45^ ^]^ To solve this problem, depositing other materials on III‐V SCs such as molybdenum disulfide (MoS_2_), and titanium dioxide (TiO_2_) has been proposed in the literature.^[^ ^45^, ^174^ ^]^

III‐V semiconductors can be utilized in PEC systems using single‐junction or multiple‐junction configurations. A single III‐V semiconductor as a photoelectrode is essentially a single‐junction photoelectrode, and multiple III‐V semiconductors in series is used as a multiple‐junction photoelectrode for increased photonic efficiency. Single junction III‐V photoelectrodes include GaAs, GaInP, InP, GaN, and InGaN. GaAs has a bandgap of 1.42 eV, making it efficient for PEC applications with its superior charge transport properties, as well as high theoretical solar conversion efficiency.^[^ ^45^ ^]^ Kang et al. utilized GaAs with an IrO_2_ overlayer to improve the OER performance of the photoelectrode, achieving a 23.1 mA cm^−2^ photocurrent density, an onset potential of 1.022 V, and a solar‐to‐hydrogen efficiency of 13.1%. GaAs/IrO_2_ photoelectrodes performed very well for stability tests as well, maintaining their operation for 192 h in an acidic environment.^[^ ^64^ ^]^ GaInP is another widely adopted single‐junction III‐V photoelectrode with a bandgap of 1.85 eV. Gu et al. worked with this material and deposited 22–26 nm thick MoS_x_ catalyst layer, and 24–30 nm thick TiO_x_ protective layer on top of it. The resulting photoelectrode exhibited 11.2 mA cm^−2^ photocurrent density, 0.4 V of onset potential, and 20 h of stability, which are not as promising like the aforementioned GaAs/IrO_2_ photoelectrode for working in acidic conditions.^[^ ^174^ ^]^

InP stands as one of the best candidates for being a PEC electrode with its 1.34 eV bandgap, which is optimum for sunlight absorption. Its low surface recombination velocity, high optical absorption coefficient, and favorable electron transfer properties renders it most favorable for PEC applications.^[^ ^45^, ^101^ ^]^ Li et al. utilized InP nanopore arrays as a photoelectrode to use it in a PEC system, which resulted in a 27.5 mA cm^−2^ of photocurrent density which is higher than that of GaAs and GaInP photoelectrodes; and with a lower onset potential than that of GaAs/IrO_2_ at 0.45 V. On the other hand, InP falls behind GaAs and GaInP material systems in terms of stability, yielding only 1.1 h of operation time.^[^ ^101^ ^]^

Multiple junction III‐V photoelectrodes aim to maximize the efficiency by utilizing different III‐V material segments (mostly 2 or 3) in a junction since each segment addresses absorption for a different spectral range, thus reducing losses and maximizing solar energy conversion.^[^ ^82^ ^]^ Varadhan et al. utilized InGaP and GaAs materials to decouple the light‐harvesting components, thus improving the overall efficiency and stability, meanwhile reducing the cost by utilizing epitaxial lift‐off (ELO) and transfer methods.^[^ ^173^ ^]^ However, the resulting photoelectrode showed 9.8 mA cm^−2^ photocurrent density and ≈0.5 V onset potential in an alkaline environment, indicating a relatively low level of performance compared to other material systems. But when stability is the major point of consideration, the InGaP/GaAs photoelectrode system operates continuously for 150 h in an alkaline environment, which constitutes an outstanding level of performance. AlOtaibi et al. coupled the III‐nitrides, InGaN, and GaN, as shown in Figure 5D–F and used parallel illumination to improve the optimization of photovoltage and photocurrent levels. The InGaN/GaN photoelectrode showed a superior 43 mA cm^−2^ of current density that is close to that of Si‐based photoelectrodes, and onset potential of 0.7 V as can be seen in Figure 5G–I.^[^ ^92^ ^]^ Lastly, to provide an example of a three junction cell, Wang et al. coupled GaInP_2_ with GaAs and Ge, and deposited a GaN protective layer on the photoelectrode to protect it from photocorrosion and oxidation, as well as to prevent the recombination of charge carriers. The resulting material had a superior onset potential of ≈2.2 V, but its photocurrent density of ≈10.3 mA cm^−2^ is not as bright as its onset potential. However, Wang et al. also measured the ABPE value of the resulting material, resulting in a value of 13.5% at ≈1.4 V_RHE_ which is very promising. Furthermore, 80 h of continuous operation is maintained by the GaInP_2_/GaAs/Ge photoelectrode, making it significantly durable against acidic environments.^[^ ^65^ ^]^ A comparison of the electrode properties for all the aforementioned material systems is provided in Table 1.

### 1.11 Transition Metal Nitrides

As non‐noble catalysts, metal nitrides (MNs) received increasing attention as OER catalysts mostly due to their higher electrical conductivity than that of metal oxides and via their unique electronic structures. Compared with most of the metal oxides, they have a bandgap between 1.9–2.5 eV, a lower overpotential, and a higher STH efficiency.^[^ ^175^, ^176^, ^177^, ^178^ ^]^ During the formation of MNs, the structure is tuned by introducing a large number of active sites through the incorporation of nitrogen atoms into the metal's interstitial sites. Based on the bonding type between parent metal and nitrogen, MNs can be classified into three categories, namely i) ionic (M: group I and II), ii) covalent (M: group III and IV), and transition metal nitrides (TMNs) where ionic, metallic, and covalent bonds are formed within a single structure, therefore possessing superior properties. They can be sub‐ or non‐stoichiometric with crystal structures of simple hexagonal, face‐centered cubic (fcc), or hexagon‐closed packed (hcp).^[^ ^179^ ^]^

Researchers have followed different approaches to improve the conductivity of the MNs and their stability by preventing the self‐oxidation of nitrogen in highly oxidative OER conditions, and the OER efficiency of the TMNs. Bandgap engineering is a well‐known and powerful strategy to improve the overall water splitting efficiency by tailoring not only the bandgap for efficient solar absorption but also the band positions to favor both water oxidation and reduction for achieving a low photocurrent onset potential.^[^ ^180^ ^]^ Creating impurity states via doping is one of the simplest approaches to tune the bandgap and band positions of a wide‐gap metal nitrides. Xiao et al. investigated the effect of Mg doping on defect formation, PEC properties, and band‐edge positions in Mg‐doped tantalum nitride photoanodes (Mg:Ta_3_N_5_).^[^ ^100^ ^]^ Their studies indicated that the gradient Mg‐doping significantly decreased the density of deep‐level defects, which is critical in suppressing defect‐related recombination, and resulted in a photocurrent density of over 8 mA cm^−2^ with improved stability of 5 h, an onset potential of 0.4 V_RHE_ and an ABPE of ≈3.25% when combined with NiCoFe‐Bi OEC.^[^ ^100^ ^]^ Interface engineering is also a preferred approach for tuning the surface properties of MNs and creating hybrid/composite structures to benefit from the synergistic effects on the PEC performances.^[^ ^90^ ^]^ Fu et al. modified the bottom interface of Ta3N5 thin film photoanode by using an n‐type In:GaN to achieve an enhanced bulk charge separation efficiency, and the top interface with a p‐type Mg:GaN for better surface charge injection efficiency. The developed In:GaN/Ta_3_N_5_/Mg:GaN heterojunction photoanode generated a photocurrent density of 9.3 mA cm^−2^ at 1.23 V_RHE_ and exhibited an ABPE of 3.46%, which was a record‐high ABPE for Ta_3_N_5_‐based photoanodes.^[^ ^90^ ^]^ Nitride‐based materials have also been used as fast hole extractors on the surface of photoanodes to improve the electron‐hole separation by reducing the recombination of charge carriers. For instance, thin layer of 2D hexagonal boron nitride (h‐BN) nanostructures have been used for the surface modification of BiVO_4_ photoanodes, and incorporated BN nanoplatelets provided ≈2.3 times higher photocurrent density than that of bare BiVO_4_. The PEC performance of the developed photoanode is further improved via dual modification using CoCr‐LDH as a co‐catalyst, leading to 3.2 times the photocurrent density of bare BiVO_4_ and a maximum photocurrent density of 3.8 mA cm^−2^ at 1.23 V_RHE_.^[^ ^93^ ^]^

Group III‐nitrides such as InGaN and GaN have also been considered as PEC electrodes. Their crystalline structure and chemical bonding between III‐Ns distinguishes them from III‐V photoelectrodes, since III‐V semiconductors have covalent bonds between their atoms, whereas III‐nitrides have ionic bonds. This makes III‐nitrides more stable in acidic and neutral electrolytes than the III‐V materials. In addition, III‐nitrides also satisfy the needs for a decent PEC operation since they have high absorption coefficients and excellent charge carrier mobilities.^[^ ^45^ ^]^ InGaN is a promising candidate for PEC applications since its bandgap is tunable between 3.4 and 0.65 eV by modulating the indium content; has high absorption coefficient, and has large carrier mobilities. Chu et al. have utilized InGaN as a photoelectrode by supporting with IrO_2_ deposited as a co‐catalyst on top; however, the resulting material exhibited a 10.9 mA cm^−2^ of photocurrent density and a 0.1 V onset potential, which are not as impressive as the previous materials. On the other hand, a high IPCE value of 93% at 440 nm wavelength and an ABPE value of 3.6% at ≈0.73 V_RHE_ were also demonstrated. Furthermore in terms of stability, InGaN/IrO_2_ photoelectrodes maintained their continuous operation for 10 h in acidic conditions.^[^ ^61^ ^]^ GaN is also utilized in PEC applications, but the high defect density in GaN films renders those materials unable to maintain a sufficient level of current for the water electrolysis reaction in PEC systems.^[^ ^45^ ^]^ Therefore, GaN is used mostly in building multiple‐junction photoelectrodes, as well as a protective layer for photoelectrodes to prevent their degradation.

### 1.12 Concluding Remarks on the Material Design

PEC electrode materials have been developed using a wide range of semiconducting materials, ranging from traditional transition metal oxides and chalcogenides to Si and group III‐V semiconductors. The use of single semiconducting materials has not been practical due to the requirements of PEC technologies. The utilization of different materials in heterostructures, modification of surface structures, and the addition of different dopants and catalysts are amongst the strategies followed by many researchers to improve their performance. Transition metal oxides are advantageous with their great abundance, non‐toxic properties, low cost and facile preparation, but their low stability in acidic/alkaline environments and high charge carrier recombination rates lead to lowering their efficiencies. On the other hand, transition metal chalcogenides can provide higher efficiencies than metal oxides when incorporated into heterostructures; their availability in different architectures and crystal structures enable researchers to tune their photoelectrocatalytic properties. However, the complex methods of synthesis involved, and their low stability hinders their applicability for PEC.

Si is very promising when used as an anode material; It is one of the highest abundant materials in nature and can provide very high current density. However, its low stability in acidic and alkaline environments hinders its usage in PEC. A variety of coating strategies can be applied as a form of protection in such environments. Finally, group III‐V semiconductors have shown great performance via tuning their photocatalytic properties, including light absorption over a wide range of wavelengths and excellent charge carrier transport properties. Significant efforts are undertaken to develop different combinations of heterostructures and device designs aiming at increasing their efficiency, however, the high cost involved in fabrication and low stability remain to be the major obstacles for their widespread commercial utilization.

### 1.13 *Operando* Characterization Techniques

While materials development including chemistry and the type of thin films or layers based on theoretical design is crucial in the development of PEC technologies, gaining a thorough understanding of the underlying mechanisms of reactions and phenomena is required. This is quite challenging due to the complex nature of the PEC processes. An understanding of the working mechanisms and degradation pathways would enable better “materials by design” toward PEC technologies that would be economical for scaled up utilization. Therefore, advanced characterization techniques are in high demand for improving the PEC technologies.

While standard characterization techniques such as XRD, XPS and electron microscopy, provide limited insight into the materials' behavior under actual operating conditions, in situ*/operando* methods have emerged as promising approaches to investigate the PEC processes under real‐time conditions.^[^ ^182^, ^183^, ^184^ ^]^ *Operando* techniques allow for the observation of materials' behavior under actual operating conditions of PEC systems, providing a more comprehensive understanding of the various mechanisms involved and the performance of materials used.^[^ ^185^, ^186^ ^]^ We expect that *operando* methods in PEC research will accelerate the development of efficient and sustainable hydrogen production technologies.

*Operando* characterization refers to measurements of PEC systems under real‐time electrochemical operation conditions. Unlike ex situ characterization, which is achieved after the disassembly of devices, *operando* techniques are powerful in providing information regarding the dynamic processes occurring during operation reflecting the true nature of the system. *Operando* techniques are distinguished for their high spatial and temporal resolution which allows for the detection of metastable states and configurations along the relaxed states.^[^ ^187^, ^188^ ^]^ The detection of such states provides invaluable information regarding the working mechanisms of PEC and degradation pathways.

Several recent studies have delivered important findings which help in directing the research path of new materials and implementing the correct approach for improving device performance. Findings show that both the material combinations and morphology of the electrodes play an important role in PEC technology performance.^[^ ^189^ ^]^ The crystal structure and morphology of a photocatalyst are important for its fundamental properties including the bandgap, Fermi level, and surface functional groups, for which more PEC‐specific *operando* characterization techniques are available. For example, in studying the spatial collection efficiency (SCE), the fraction of charge carriers is measured at a specific position which leads to generating a photocurrent.^[^ ^190^ ^]^ Another specific technique called optical pump‐probe spectroscopy is utilized for measuring the optical properties of PEC materials and events resolved in ultrafast time scales ranging from femtoseconds (fs) to nanoseconds (ns), where laser light is pulsed and the absorption spectrum is obtained with a time delay.^[^ ^191^ ^]^ Femtosecond transient absorption technique has been applied in the study of the electronic transitions, vibrational relaxations, and charge carrier dynamics. By measuring the changes in absorption at different wavelengths and time delays, researchers can determine the lifetimes of excited states, identify intermediate species, and track energy transfer processes.^[^ ^192^, ^193^, ^194^ ^]^ Examples for studying the charge transfer dynamics in metal oxide transition metals such as BiVO_4_ revealed the relaxation and trapping rates for electrons and holes which showed no effect by applying the voltage bias.^[^ ^195^ ^]^ In another study, the charge carriers in the heterojunction WO_3_/BiVO_4_ was found to be strongly affected by the application of an external bias as a consequence of the electric field built in at the WO_3_/BiVO_4_ heterojunction.^[^ ^196^ ^]^ Another example can be mentioned for the elucidation of the low H_2_ evolution in Ta_3_N_5_ compared to O_2_ evolution which was found to be from the absence of electron transfer to the cocatalyst by the virtue of electron trapping process.^[^ ^197^ ^]^

Undoubtedly the most common *Operando* technique used for PEC characterization is XAS. In the following section, we will review selected important findings of spectroscopic *operando* characterization techniques including XAS, XPS, IR, Raman, and EIS.

### 1.14 X‐Ray Absorption Spectroscopy (XAS)

X‐ray absorption spectroscopy (XAS) utilizing synchrotron radiation proves to be a highly efficient technique for acquiring direct *operando* information regarding the chemical environment and electronic structure. The tunable nature of synchrotron sources enables XAS to provide element‐specific information.^[^ ^198^ ^]^

The XAS spectrum can be classified into two categories depending on the absorption threshold: X‐ray absorption near‐edge structure (XANES) and extended X‐ray absorption fine structure (EXAFS). In XANES, the spectrum typically spans from the edge to ≈50 eV above the threshold, offering insights into the oxidation state of the system.^[^ ^199^ ^]^ Conversely, EXAFS encompasses a spectrum beyond the XANES limit, extending up to 1000 eV above the threshold, providing valuable information such as bond length and coordination.^[^ ^199^, ^200^ ^]^

Typically, X‐ray sources can be categorized into two types: high‐energy X‐rays known as hard X‐rays, and low‐energy X‐rays referred to as soft X‐rays. Soft XAS finds utility in examining the K‐edge of lighter elements such as carbon, oxygen, and nitrogen. Moreover, it is also capable of detecting the L‐edge (2p to 3d excitation) of 3d transition metals like iron, cobalt, nickel, and titanium.^[^ ^201^ ^]^ However, due to the limited X‐ray penetration depth associated with soft XAS, alternative approaches such as fluorescence methods are employed.^[^ ^202^ ^]^

With XAS, electronic information can easily be obtained. Moreover, elements at low concentrations are within the detection limit. Being able to detect charge transfer dynamics makes this tool very effective for PEC applications. Operational conditions and some key findings of XAS studies for PEC applications are tabulated in **Table** **2**.

**Table 2:** Operational Conditions and Findings of XAS studies for PEC.

System/Substrate/Reaction	X‐Ray Source	Spectral Edge	Operational Conditions	Findings	References
WO_3_/ FTO/ OER in a Na_2_SO_4_ electrolyte	LISA beamline at European synchrotron radiation facility (ESFR), using a Si(311) double crystal monochromator, Pd mirrors with a cut‐off energy of 20 keV	W LIII‐edge XANES	OCV, 0.35 and 1 V in UV–vis under light and dark conditions	‐ The empty states are t_2g_ and e_g_ ‐ After illumination, the t_2g_ band was filled within the first few seconds ‐ A structural rearrangement occurs on a longer time scale	[50]
Mn_3_O_4+δ_ coated Fe_2_O_3_/FTO/OER in 1.0 M NaOH electrolyte	SSLS XAFCA beamline. The X‐ray beam energy was calibrated using the Fe metal foil K‐edge at 7.112 keV	XANES spectra on Mn and Fe K‐edges	0.5 to 1.8 V (V_RHE_) under light illumination.	‐ Mn^3+^/Mn^4+^ pair is responsible for the OER	[209]
NiO_x_ coated Fe_2_O_3_/FTO/OER in 1.0 M NaOH electrolyte	LISA beamline at ESFR, using a Si(311) double crystal monochromator, Pd mirrors with a cut‐off energy of 20 keV	XANES spectra on Ni K‐edge	0.5, 0.7, and 1.3 V in dark and under LED illumination	‐ Improved photocurrent due to the rapid transfer of generated holes	[51]
CoBx coated Fe_2_O_3_/FTO/OER in 0.1 M sodium borate electrolyte	KMC_2_ beamline at the BESSY II synchrotron	XANES Co K‐edge	OCV and 1.73 V in light and dark conditions	‐ The holes migrated from the hematite oxidize Co atoms and passivate the surface traps	[225]
ZnO/Fe_2_O_3_ core‐shell nanowires/FTO/OER in 1.0 M NaOH	BL20A and BL17C beamlines at the National Synchrotron Radiation Research Center in Hsinchu, Taiwan and 4U beamline at the Ultraviolet Synchrotron Orbital Radiation facility in Okazaki, Japan	XAS spectra of O K‐edge, Zn L_3_,2‐edge, and Zn K‐edge and STXM‐XANES spectra O K‐edge	Bare and Fe_2_O_3_ coated surfaces under dark and photo illuminated conditions	‐ Coating reduced the traps on the surface which are recombination centers	[232]
Ir/IrO_x_/ p^+^n‐Si and IrO_x_/Au/p^+^n‐Si/OER in 1 M H_2_SO_4_	Stanford Synchrotron Radiation Light source	XANES spectra of Ir LIII‐edge,	1, 2 and 3 nm IrO_x_ coating under irradiation	‐ Highest oxidation state is reached when the coating is thin	[52]

Since in XAS the penetration depth is in the order of µm, the technique averages the properties of the related atoms. Nevertheless, it is a powerful technique to understand the properties of the atoms under *operando* conditions.^[^ ^203^, ^204^ ^]^ Since the lifetime of photogenerated charges is short, preparing a catalyst layer with a very high surface to bulk ratio can be effective but this cannot entirely satisfy *operando* conditions. Another alternative is to perform the characterization under dark and illuminated conditions and obtain the difference under these states. Fracchia et al. used this method on WO_3_ which is a very common photoanode material.^[^ ^50^ ^]^ In this study, the WO_3_ layer was obtained by spin coating H_2_WO_4_ colloidal suspension on FTO and annealing it under air. The PEC OER characterizations were performed in a Na_2_SO_4_ electrolyte. During the XAS measurement, the W‐L_III_ edge was acquired. It was observed that the empty 5d states consist of split t_2g_ and e_g_ crystal field symmetries.^[^ ^205^ ^]^ Under illumination, a significant intensity change was observed in the t_2g_ symmetry due to the filling of this state. While this phenomenon occurs within the first second, they also observed a structural rearrangement of the material that happens in a longer duration.^[^ ^206^ ^]^

α‐Fe_2_O_3_ is a very common and low bandgap (1.9–2.2 eV) semiconducting material with a theoretical maximum photocurrent density value of 12.6 mA cm^−2^ at AM 1.5G solar spectrum.^[^ ^207^, ^208^ ^]^ However, only a small portion of this value can be obtained due to charge transport properties. Depositing α‐Fe_2_O_3_ as nano‐sized particles can decrease electron/hole recombination. Nevertheless, nanosizing still cannot push the current density values close to the theoretical levels. To achieve that, some co‐catalysts like MnO_x_ are used on top of α‐Fe_2_O_3_. Xu et al. performed an *operando* investigation of manganese oxide‐based (Mn_3_O_4+δ_) on a hematite (α‐Fe_2_O_3_) surface for a PEC photoanode system to answer the debates of whether Mn is effective in the PEC process or not.^[^ ^209^ ^]^ The electrodes were prepared by first electrodeposition of iron oxide species on FTO and annealing then dip coating in a manganese salt with final annealing. In situ XAS testing was performed in a basic electrolyte with a three‐electrode PEC cell under varying potential and an inert atmosphere. Between the potential values 0.5 to 1.8 V_RHE_ under illumination, XAS characterizations reveal that the Mn oxidation state gradually increases from +2.8 to +3.5. On the other hand, the oxidation state of Fe stays the same at the value of +3 all through the potential window. Having a constant oxidation state for Fe and a value of +3.5 for Mn at 1.8 V indicates that the Mn^3+^/Mn^4+^ pair is responsible for the OER reaction. In parallel to this result, the PEC tests also reveal that Mn_3_O_4+δ_ utilizes the charges more effectively. While the effect under 1.2 V is negligible, above that potential, Mn becomes dominant and reaches maximum performance at 1.7 V with a +3.4 oxidation state.

**Figure 6:**
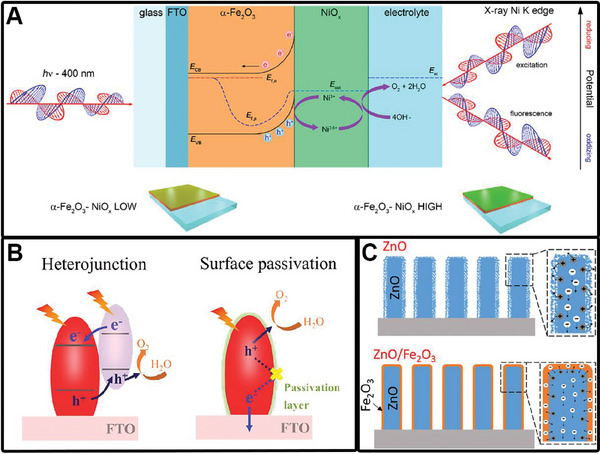
Applied Operando XAS studies fo PEC electrodes A) Schematic representation of operando X‐ray/photoelectrochemical study on the NiOx coated Fe_2_O_3_ photoanode. Reproduced with permission.^[^ ^51^ ^]^ Copyright 2020, ACS Publishing. B) Illustration showing the effect of surface modification enhancing the performance. Reproduced with permission.^[^ ^239^ ^]^ Copyright 2020, ACS Publications. C) Surface engineered Fe_2_O_3_ nanowires coated with ZnO showing the recombination in the traps. Reproduced with permission.^[^ ^232^ ^]^ Copyright 2020, Elsevier.

Covering fewer stable semiconductors with a catalyst layer helps improve charge separation.^[^ ^210^, ^211^ ^]^ Nevertheless, the mechanism of this improvement is generally not clear. Some of the mechanisms are charge transfer between the interface and the passivation of recombination centers.^[^ ^212^, ^213^, ^214^, ^215^, ^216^, ^217^ ^]^ Studies show that the catalyst layer acts as a hole collector due to effective band bending.^[^ ^137^, ^218^ ^]^ In another study, in situ XAS was employed again to understand the co‐catalyst mechanism in an α‐Fe_2_O_3_/NiO_x_ system.^[^ ^51^ ^]^ The electrodes were prepared by a similar technique with MnO_x_ but the authors also studied the thickness of the overlayer (**Figure** **6**A). In situ tests were also performed under alkaline conditions. The Ni K‐edge of photoanodes was first probed under darkness. The results showed that through the potential window of 0.5−1.3 V, the oxidation state of Ni stayed at +2. On the other hand, under the illumination of visible light after 0.7 V, the oxidation state increased to +2.45 and then to +3.4 at the highest potential. The photoanode with NiO_x_ co‐catalyst overlayer showed an improved photocurrent. This improvement was due to the rapid transfer of holes generated in α‐Fe_2_O_3_ and the promotion of the NiO_x_’s oxidation state.

On hematite, as an OER overlayer co‐catalyst, many different materials like oxides, hydroxides, and phosphates were implemented.^[^ ^219^, ^220^, ^221^, ^222^, ^223^ ^]^ A more recent material type is cobalt borate (CoB_x_), which is reported to be more active than cobalt phosphates.^[^ ^224^ ^]^ Xi et al. performed an in situ XAS analysis of CoB_x_ on a hematite PEC anode.^[^ ^225^ ^]^ The CoB_x_ overlayer was deposited on the FTO/α‐Fe_2_O_3_ electrode by a photo‐electrodeposition method and the PEC electrolyte was basic. The XAS data were obtained at the K edge of Co and the oxidation state of Co, at OCV, without hematite sublayer was 2.89. This result is consistent with the literature.^[^ ^226^ ^]^ However, the interesting thing is when hematite is present, at OCV, the oxidation state rises to 3.38. This was explained by the photoelectrodeposition method used for the CoB_x_ layer. It was argued that during the deposition holes migrated from the hematite layer and oxidized Co atoms and passivate the surface traps (Figure 6B). Another important observation is that even without applying a potential, the oxidation state of Co increased from 3.38 to 3.49 under illumination. This helps a better charge separation. At higher potentials, a better band bending occurs which enhances this effect.

For PEC applications the photoactive material should have a suitable bandgap for effective sunlight absorption. Equally important, the charge carriers should have a long diffusion length, and the band edges must be aligned with the water oxidation and reduction reactions. Some of the stable metal oxide materials that can be used for this purpose are ZnO and TiO_2_.^[^ ^227^, ^228^ ^]^ Nevertheless, they are wide bandgap semiconductors and cannot effectively use sunlight. One way to overcome this issue is combining these materials with low bandgap materials and forming a heterojunction.^[^ ^72^, ^229^ ^]^ One of these combinations is Fe_2_O_3_/ZnO.^[^ ^230^ ^]^ ZnO is a cheap direct bandgap semiconductor, wherein a 1D structure has a fast charge carrier property.^[^ ^231^ ^]^ Lu et al. prepared Fe_2_O_3_/ZnO PEC anodes by first growing ZnO nanowires on FTO, then spin‐coating an iron solution onto the substrate.^[^ ^232^ ^]^ After annealing, a core‐shell structure was obtained. The PEC characterization was performed under basic conditions and O K‐edge, Zn L_3,2_‐edge, and Zn K‐edge XAS spectra were examined. Zn L‐edge shows the electron transitions from the core levels (Zn 2p) to the hybridized states (4s/3d).^[^ ^233^ ^]^ Upon illumination, this spectrum did not essentially change, revealing that these orbitals are not active for the OER reaction. On the other side, the Zn K‐edge's (1s‐to‐4p) intensity dropped upon illumination, showing the 4p is the active orbital.^[^ ^234^ ^]^ In parallel to that, the Fe L‐edge intensity increased during the same process. This means that there is an electron transfer from Fe_2_O_3_ to ZnO. During the absorption of the photons, electrons and holes are generated. While the electrons are transferred to the counter electrode, the holes move to the anode surface to generate O_2_. When ZnO oxide is not coated with Fe_2_O_3_, there are plenty of defects that are centers for electron‐hole recombination (Figure 6C). However, after coating the core‐shell structure has a better crystallinity and the recombination is reduced.

Silicon is a promising electrode material for PEC applications since it is already used in light‐harvesting applications. Nevertheless, silicon is not stable under aqueous conditions. To improve the stability, some active or passive layers are needed.^[^ ^235^, ^236^ ^]^ In the literature, for the OER, usually alkaline condition‐based materials are studied since there is not much material to study under acidic conditions. IrO_x_ is one of those materials that have high activity and stability in acidic electrolytes.^[^ ^237^ ^]^ There are several studies related to IrO_x_’s performance, however, their results are not very consistent. It is argued that this discrepancy can be related to the low resolution of XAS characterization for Ir. Li et al. studied IrO_x_ performance with high energy resolution fluorescence detection XAS (HERFD XAS) under an acidic environment.^[^ ^52^ ^]^ The PEC anode was p^+^n‐Si and sputter‐deposited with Ir/IrO_x_ film. They performed their *Operando* characterization with film thickness values of 1, 2, and 3 nm. For every electrode, the oxidation state increased with increasing potential. However, the increase was more pronounced in thinner films. For example, for the 3 nm film, the oxidation state reaches 3.74 V, while for the 1 nm film, it is ≈4.00 V. Interestingly, for the 1 nm film, the oxidation state started to decrease after 1.46 V (V_RHE_). This was explained by the situation that the highest oxidation state is reached when the surface is fully covered with O atoms below the onset OER.^[^ ^238^ ^]^ After passing the OER onset potential, the surface releases O_2_ molecules, and the oxidation state starts to decrease. This effect cannot be observed with thicker films, and it shows the importance of the overlayer thickness.

### 1.15 X‐Ray Photoelectron Spectroscopy (XPS)

Although there are a number of *Operando* XAS studies for PEC, *Operando* XPS for PEC has been used in a very limited number of studies in the literature. This is due to the high vacuum requirement in XPS which is hard to sustain for the PEC systems. In one example study, Yano and Sharp investigated the OER reaction mechanism on top of a biphasic CoOx catalyst using *Operando* XPS.^[^ ^30^ ^]^ In a 3‐electrode system configuration and under conditions set to match that of OER, the system included a catalyst working electrode, a Pt foil as counter electrode and Ag/AgCl as reference electrode. The cell was set under basic conditions (1 m KOH), 18 Torr, and room temperature. *Operando* XPS analysis was done to trace the O_1s_, Co_2p_, and the valence band spectra at various operating voltage conditions. The XPS result revealed the presence of two phases within the catalyst layer. The formation of the CoO(OH) phase was tracked as a function of applied bias potential. It was revealed that the layered CoO(OH) phase serves in enhancing the OER kinetics of the catalyst layer.

### 1.16 Infrared and Raman Spectroscopy

Infrared (IR) and Raman spectroscopy techniques are used to distinguish reaction intermediates and to identify the possible reaction mechanisms during PEC. The main advantage of IR and Raman spectroscopy techniques is their sensitivity to the functional groups formed during the photocatalytic reactions. Complication in PEC catalysts arises in the short lifetime of the intermediates and their minimal quantity at the surfaces of the PEC catalysis.^[^ ^240^ ^]^ The main requirement needed is to perform the analysis during the illumination of the photocatalyst in a very fast time‐scale to catch the intermediates formed. **Table** **3** summarizes *Operando* Photo/Raman and IR studies used to investigate the intermediates formed during photoelectrochemical water splitting of different catalysts.

**Table 3:** Summary of the Operando IR and Raman Spectroscopic Studies for Photocatalytic Water Oxidation.

Technique	Studied System /Active Substrate/Reaction	Excitation Source	System Conditions	Measurement Parameters	Variables	Findings	References
Raman spectroscopy	Transition Metal Dichalcogenides/MoS_2x_Se_2(1−x)_ nanosheets/HER	532 nm laser (5 mW)	0.5 m Na_2_SO_4_ solution	Illuminated by light source (100 mW cm^−2^)	0.10 to 0.20 V	‐Hydrogen atoms are initially adsorbed to the active S or Se atoms by chemical bonding S‐H and Se‐H to form intermediate species in MoS_2x_Se_2(1−x)_	[29]
Surface‐enhanced Raman spectroscopy	Ag−Au−Ag and Au−Ag/Ag_2_S heterojunction nanorods/hot spots temperature of the Ag−Au−Ag and Au−Ag/Ag_2_S/HER	632.8 nm from a He−Ne laser	0.5 m H_2_SO_4_ solution	Illuminated by light source (100 mW cm^−2^)	‐0.8 to ‐0.3 V (V_RHE_) Temperature range (20–100 °C)	‐Hot spots temperature of the Ag−Au−Ag and Au−Ag/Ag2S heterojunction nanorods surface illuminating for different times was detected	[55]
Rapid‐scan FTIR	IrO_2_ colloidal solution/O‐O vibrational mode at 830 cm^‐1^/OER	476 nm (300 mW)	excitation of IrO_2_ colloidal solution with a 1 s pulse	Illumination of the colloidal solution by a 476 nm laser pulse	Isotope labeling with Deuterium, Oxygen‐18	‐Intermediates for the water oxidation reaction on multi‐electron‐transfer catalysts were identified as surface hydroperoxide species	[53]
FTIR	Hematite α‐Fe_2_O_3_/FeIV = O group/OER	ATR Set‐up A 45° angle ZnSe crystal	D2O containing 0.2 m KCl	Illumination by LED ultraviolet flashlight (395 nm) (10 mW cm^−2^)	1.1 to − 2.0 V (V_RHE_) pH 6.9 and 13	‐First direct evidence of a surface Fe(IV) = O intermediate of water oxidation on hematite	[31]
FTIR	Hematite Photoanode/O−O bond formation pathway by metal oxo‐species/OER	ATR Set‐up A 45° angle ZnSe crystal	Three electrode systems with different applied potentials	Back‐illumination with a 150 W xenon lamp coupled to a filter	18 O and D isotope	‐At near‐neutral pH region O‐O bond formation occur due to the surface trapped holes ‐Metal oxo‐species and hole transfer result in the formation of superoxide species	[54]
ATR‐FtIR	Ga_2_O_3_‐based Photocatalysts/Water splitting	263 nm laser (200 mW) ATR Set‐up ZnSe crystal	0.3 g distilled water was spread on the catalyst film	Illumination by laser source: 200 mW, 1000 Hz, 263 nm	18 O and D isotope	‐New mechanism based on the direct formation of hydroxyl radicals by photogenerated holes is proposed	[48]
Photoelectrochemical attenuated total reflection Fourier transform infrared	BiVO_4_ photoanode/BiVO_4_/electrolyte interface/HER	Bruker Vertex 70 spectrometer equipped with a liquid nitrogen cooled MCT detector and a Veemax‐III ATR accessory	Three electrode configuration with a Ag/AgCl reference electrode	Solar simulator with light intensity of 6 mW cm^−2^	Open circuit voltage and applied bias	‐Preferential dissolution of V from the BiVO_4_ happens upon illumination at open circuit conditions ‐Dissolution of Bi and V is observed upon illumination under anodic potentials	[56]

One of the earliest studies was performed by Sivasankar et al. which studied the formation of the surface intermediates on Iridium oxide nanoclusters during water oxidation.^[^ ^53^ ^]^ During electrochemical measurement of water oxidation upon illumination with a 476 nm laser pulse, they used rapid‐scan FTIR to detect the intermediates formed. An ATR accessory was used that is 3 mm in diameter with a diamond plate that has three reflections. The IrO containing aqueous solution was put on the top of the plate. They followed the formation and depletion of O−O and O−H vibrational bands with isotope labeling which enabled the identification of the surface intermediate as a hydroperoxide (IrOOH) species. A mechanism was proposed which suggests the formation of an O−O bond by the reaction of an Ir(V) oxo intermediate with water or hydroxyl group.

Similar following studies were conducted for investigating the formed intermediates on hematite (α‐Fe_2_O_3_) during water photoelectrochemical oxidation. For example, Zandi et al. studied the oxidized surface states that mediate PEC water oxidation on haematite electrodes utilizing ATR‐IR spectroscopy.^[^ ^31^ ^]^ They also used isotope labeling to determine the formed species during the processes. The result shows the first oxidation reaction to form an iron‐oxo group on the surface of the hematite by valence‐band holes. Following that, Zhang et al. investigated the oxo formation and subsequent rate‐limiting O−O bond formation mechanism on hematite.^[^ ^54^ ^]^ Using isotope labeling at different pH environments, they proposed the formation of multiple surface Fe = O species upon the increase of the pH (10–12). This also leads to the formation of O−O bond which was found to result in faster water oxidation kinetics without accumulation of stable intermediates. Whereas, at higher pH (≥13) a different mechanism takes place that is dependent on coupling the neighboring metal oxo‐species.

Other than hematite, Ga_2_O_3_ based photocatalysts for water oxidation were investigated by Chen et al. using *Operando* FTIR spectroscopy coupled with Mass Spectroscopy.^[^ ^48^ ^]^ They proposed a different mechanism than what is observed for other semiconductor photocatalysts. The mechanism is suggested to form direct hydroxyl radical during water oxidation. Their investigation involved Rh_0.5_Cr_1.5_O_3_ loading and Zn doping which showed higher photocatalytic activity. Isotope labeling for hydrogen and oxygen was used and revealed the direct hydroxyl radical formation mechanism which was supported by the observation of no participation during the reaction of the lattice oxygen in the catalyst.

**Figure 7:**
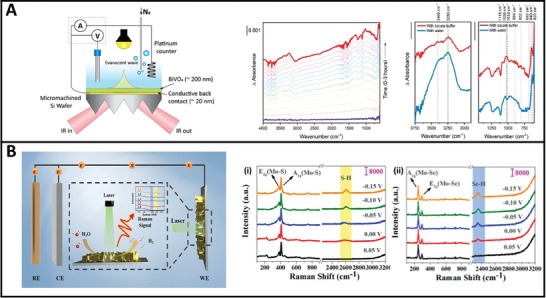
Operando FTIR and Raman studies for PEC A) Schematic of operando ATR‐FTIR used to study photocatalytic water oxidation with IR spectra of the BiVO_4_/electrolyte interface acquired over the course of photocharging in the case of a borate buffer electrolyte. Reproduced with permission.^[^ ^56^ ^]^ Copyright 2021, ACS Publications. B) Schematic of the photoelectrochemical system with Raman analysis with operando Raman spectra of (i) MoS_2_, (ii) MoSe_2_ in the voltage of 0.05, 0.00, −0.05, −0.10, and −0.15 V. Reproduced with permission.^[^ ^29^ ^]^ Copyright 2020, Wiley.

Venugopal et al. utilized *Operando* photoelectrochemical ATR to reveal the dynamic nature of semiconductor−electrolyte interface in BiVO_4_ multinary metal oxide photoelectrodes.^[^ ^56^ ^]^ Their investigation was made under illumination at open circuit voltage as well as applied bias. The scheme of the set‐up and the spectra collected are shown in (**Figure** **7**A). They revealed that under illumination different metal dissolutions are taking place depending on the applied potential. The difference in the dissolution rates alters the metallic surface ratio and results in a heterojunction between the surface and the pristine bulk structure. This causes a significant improvement in band bending near the interface. They suggested the direct responsibility of the band bending for the improvement in the photocatalytic performance by the photocharging treatment.

Besides IR spectroscopy, *operando*‐Raman was also used to investigate the formed intermediates during photocatalytic water oxidation. Guo et al. employed linear‐sweep voltammetry with the *operando*‐Raman spectroscopy set‐up that is shown in (Figure 7B). They intended to reveal the photocatalytic HER intermediates of multielement photocatalysts (MoS_2x_Se_2(1−x)_) and to assess their photocatalytic performance.^[^ ^29^ ^]^ Their investigation revealed, as can be seen in the spectra presented in (Figure 7B), that hydrogen atoms are initially adsorbed to the active S or Se atoms followed by the oxidation reaction.

In another study, Wang et al. used surface‐enhanced Raman spectroscopy to measure the hot spots temperature of nanostructure surfaces instantly during photocatalytic reactions.^[^ ^55^ ^]^ They utilized N‐C stretching vibration adsorbed on the nanostructures which shows linear variation with the surface temperature. It was used to study the hot spots of Ag−Au−Ag and Au−Ag/Ag_2_S heterojunction nanorods under illumination which were found to be 85 and 80 °C when illuminating for 30 min.

These examples show how *operando*‐IR/Raman technique can provide valuable information regarding the mechanisms of photoelectrocatalytic water oxidation. The most powerful properties are in the ability to monitor the formed intermediates along with the reactants and products during the extent of the photoelectrocatalytic reaction.

### 1.17 Electrochemical Impedance Spectroscopy (EIS)

EIS is employed, in a limited number of studies, to reveal kinetic and mechanistic aspects of charge and mass transfer of PEC systems. *Operando*‐EIS is used to study the change in the charge transfer resistances and charge carriers’ behavior under illumination of the photocatalyst and/or with applying bias to match operating conditions. The *Operando*‐EIS data is collected and fitted to equivalent circuit elements to observe the variation in the parameters as potential changes.

**Figure 8:**
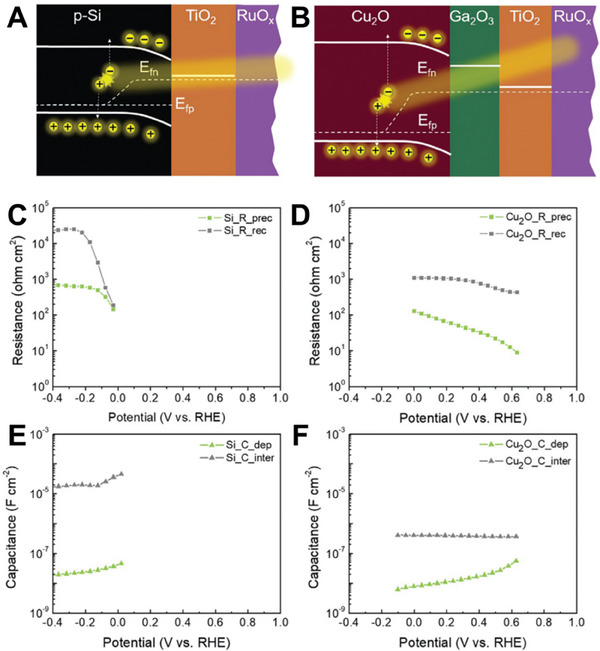
Schematics of the device and obtained Operando‐EIS data of A) Si and B) Cu2O photocathodes. C,D) Resistances and E,F) Capacitances associated with the different absorber materials obtained by fitting the EIS data of each multi‐layered photocathode after the photocurrent onset. Reproduced with permission.^[^ ^28^ ^]^ Copyright 2021, Wiley.

**Table** **4** summarizes the studies which utilized *Operando*‐EIS for photocatalytic systems. For instance, Moehl et al. developed a resistance‐based method utilizing EIS to identify the underlying potential‐dependent processes of photocathodes of various layers under operating conditions.^[^ ^57^ ^]^ Their study was performed on Cu_2_O/Ga_2_O_3_/TiO_2_/RuO_x_ and p–Si/TiO_2_/RuO_x_ photocathodes aiming for elucidating charge transport limitations and revealing the problematic interfaces, (**Figure** **8**A,B). The constructed photocathodes were studied under dark and illumination conditions and EIS was measured by changing the voltage between −0.2 to 1.2 V. The obtained EIS data were fitted to equivalent circuit elements and the charge transfer resistances were assigned to the related photophysical or electrochemical processes then evaluated with the potential change as can be seen from their result in (Figure 8C–F). The method developed was used to examine the surface quality of the photocathodes. It helped in identifying that interface defects limit the charge transfer between the Cu_2_O and the buffer layer. Moreover, they were able to identify a potential barrier at the Ga2O_3_/TiO_2_ interface for the photogenerated electrons. This potential barrier was shown to limit the increase of the photocurrent at the onset of the hydrogen evolution.

**Table 4:** Operando‐EIS studies for water PEC systems.

Studied system/Active substrate/Reaction	Excitation AC + Applied bias	Frequency range	Test Conditions	Extracted parameters	Findings	References
Multilayer photocathode devices/p‐Si/TiO_2_/RuO_x_ and Cu_2_O/Ga_2_O_3_/TiO_2_/RuO_x_/HER	AC: Not specified DC: −0.2–1.2 V (V_RHE_)	1 MHz–0.2 Hz	In the dark and under illumination	Charge transfer resistance	‐ Charge transfer between the Cu_2_O and the buffer layer is limited by the interface defects ‐ Potential barrier was identified at the Ga_2_O_3_/TiO_2_ interface for the photogenerated electrons	[57]
TiO_2_ coated Sb_2_Se_3_ photocathodes and Cu_2_O and Si photocathodes/Sb_2_Se_3_, Cu_2_O and Si/HER	AC: 15 mV DC: −0.2–0.55 V (V_RHE_)	7 MHz–0.1 Hz	In the dark and under illumination	Charge transfer resistance	‐ The photogenerated carriers possess higher lifetime in Sb_2_Se_3_ and Cu_2_O photocathodes than the Si photocathodes	[28]
Atomic layer deposited TiO_2_/TiO_2_ /HER	AC: 15 mV DC: 0.4–1.0 V (V_RHE_) steps of 50 mV with 60s equilibrium time	1 MHz–0.1 Hz	1.0 m KOH and 0.5 m H_2_SO_4_ solutions	Charge transfer resistance	‐Charge transfer through the TiO_2_ layer did not show a clear thickness dependence ‐Charge transport mechanism is conduction band‐based	[58]
CuO Nanowire Array Photocathodes/CuO/HER	AC: 10 mV at open circuit voltage	1 MHz–0.1 Hz	455 nm LED illumination (36 mW cm^−2^)	Charge transfer resistance, Double layer capacitance and Warburg coefficient	‐Unsuitable distorted CuO arrays are with a much higher recombination rate	[241]

In similar studies following the same method, Yang et al. performed the analysis on different semiconductor multilayer materials.^[^ ^28^ ^]^ TiO_2_‐coated Sb_2_Se_3_ photocathodes were analyzed, and EIS responses were assigned to the corresponding processes. Then the analysis was extended to crystalline Si and Cu_2_O substrates. They demonstrated the direct comparison of the minority carrier lifetimes of each device under potential change. Their analysis showed that Si photocathodes have longer lifetimes than Sb_2_Se_3_ and Cu_2_O photocathodes. Likewise, Cui et al. studied atomic layer deposited TiO_2_ photoanodes with various thicknesses.^[^ ^58^ ^]^ Their work suggested that the charge transfer through the TiO_2_ layer did not show a clear thickness dependence and the charge transport mechanism is a conduction band‐based transport mechanism despite the presence of defect states in the bandgap of TiO_2_.

In a different study, Cao et al. investigated the charge transport and transfer kinetics for CuO nanowire arrays photocathodes utilizing photoelectrochemical impedance spectroscopy.^[^ ^237^ ^]^ EIS measurements were obtained at the open circuit voltage of CuO nanowire arrays photocathodes in the presence and absence of 455 nm LED illumination. The EIS data was fitted with equivalent circuit elements and evaluation of the parameters variation with the anodization rates in preparation was assessed. Their findings suggested that unsuitable geometric features of the nanowire arrays, such as inhomogeneous diameters, sparse distribution, and short lengths, may lead to a serious recombination issue within CuO structures and distorted arrays possess much higher recombination rate.

These studies show the power of the *operando*‐EIS technique to determine important kinetic aspects for the evaluation of the photocatalytic anodes. It reveals, along with the limiting factors, the electrochemical parameters that can be improved for better photocatalytic performance.

### 1.18 Challenges and Future Directions

The cost of H2 from water electrolysis is ≈6.22 and 8.43 $/kg H_2_ from PEC at a similar efficiency of 10%. The techno‐economic benefits from that type of system over water electrolysis are still not clear and restricted even in the perfect scenario. The cost of the H_2_ needed to decrease but the membrane and the photoactive material used in the PEC system have a considerable influence on the costs, with 1.50 and 1.35 $/kg H_2_ respectively.^[^ ^15^ ^]^ Moreover, the U.S. Department of Energy's Hydrogen and Fuel Cell Technologies Office has set solar‐to‐hydrogen efficiency to 25% as the ultimate target through PEC water‐splitting. Even with the perfect scenario, it is not possible to achieve such a high efficiency without using cost‐effective materials. The imperative task for achieving mass production of PEC components and materials holds paramount importance in the endeavor to lower the cost of H_2_ production. Mass production of components and materials allows for the realization of economies of scale, which, in turn, yield significant cost reductions. In the context of a PEC system, ≈55% of the total cost (4.61$/kg H_2_) can be attributed to the module which also encompasses all of the active materials. By dedicating efforts to achieving mass production, both academic researchers and manufacturers can further yield innovative synthesis methodologies, enhanced material properties, along with a significant reduction of manufacturing expenses. As a consequence, the cost of photoactive materials, presently standing at ≈1.35 $/kg H_2_, can be considerably diminished.^[^ ^242^ ^]^ A novel approach involves integrating PEC hydrogen production with wind turbines to provide a consistent renewable energy supply, mitigating the intermittency of both sources. Hydrogen produced via PEC can be used as a storage medium for excess energy generated from wind and solar, helping to balance supply and demand. Nevertheless, the overall stability of state‐of‐the‐art photoelectrodes is still not adequate due to their chemical instability, electrolyte resistance, mass transfer problems, and unoptimized experimental set‐up or design.^[^ ^87^ ^]^

PEC technology is complex and requires different features to be met in one material. The four main requirements are high solar absorption, efficiency of charge separation, fast surface reactions, and high chemical stability. A semiconducting material meeting all the requirements is yet to be found. However, strategies for enhancing efficiency and stability are what the scientific community is pushing to develop. Hybrid approaches and combination of different materials can deliver satisfactory performance. Strategies in materials design including heterojunction formation and bandgap engineering by combining different semiconducting materials, doping, and applying different coating materials are shown to greatly enhance the performance.

### 1.19 Novel Material Implication

The introduction of new materials that can provide high current density with satisfactory stability is essential for developing applicable and affordable PEC technology. The high current density materials are limited to Si and group III–V semiconductors; however, they suffer from low stability which can be mitigated by coating them with protective layers as shown in the previous sections of this review. Protective layers including metal oxides, thin metallic layers, and novel materials such as MXene have led to great enhancements in stability, and further developments in this area could provide better enhancements.^[^ ^243^, ^244^ ^]^

Moreover, new semiconducting materials should be investigated for their applicability in PEC technology. An example is the utilization of organic semiconductors for which recent research has shown promising results.^[^ ^245^, ^246^, ^247^, ^248^ ^]^ A major advantage that organic semiconductors bring about is the ability to tune their structures to meet the requirements of PEC. Further investigation of suitable organic semiconductors has the potential to further enhance the efficiency of PEC devices with lower cost.

### 1.20 *Operando* Characterization

*Operando* techniques most effectively contribute to understanding the underlying mechanisms, formed intermediates, and the degradation pathways of PEC. *Operando* XAS and XPS can shed light on the behavior of PEC materials and identify potential degradation mechanisms, leading to more robust and durable materials. *Operando* IR and Raman are excellent in determining the forming of the intermediates during the working of the photocatalysts. *Operando* EIS is informative for extracting the charge and mass transfer properties of the PEC material.

Looking ahead, there are several exciting prospects for *Operando* characterization in PEC. First, advancements in instrumentation and experimental setups will enhance the spatial and temporal resolution, enabling finer characterization of the dynamic processes involved in H_2_ production. This will allow researchers to capture the intricate interplay between material properties and reaction kinetics, ultimately leading to more efficient PEC systems. Moreover, the integration of different *Operando* complementary techniques can provide a comprehensive understanding of the photoelectrochemical interface. Synergistic data obtained from these multimodal approaches will contribute to a holistic picture of the system, enabling targeted material design and optimization.

However, challenges remain in the application of *Operando* techniques in PEC H_2_ production. One key hurdle is the development of suitable sample environments that can simulate realistic operating conditions, including illumination and electrolyte flow. Replicating such environments while maintaining high‐quality signal acquisition poses technical difficulties, necessitating the design of specialized and robust experimental setups. Additionally, the interpretation of *Operando* data requires sophisticated data analysis techniques and theoretical modeling. Extracting meaningful information from the complex spectra, assigning spectral features to specific electronic and chemical states, and correlating them with device performance metrics demand advanced computational approaches like machine learning and artificial intelligence.

In future applications, the incorporation of *Operando* techniques into large‐scale reactors and the translation of laboratory‐scale findings to industrially relevant systems will require close collaboration between researchers and industry partners. The combination of improved experimental techniques, integration with complementary methods, and sophisticated data analysis approaches will pave the way for the development of efficient and sustainable green hydrogen technologies.

### 1.21 Computational Studies

Developing accurate models using computational methods such as the Density Functional Theory (DFT) can play a crucial role in deeper understanding of the underlying mechanisms and a thorough investigation of potential degradation issues, complementing experimental data and providing valuable insights into the fundamental processes involved in PEC hydrogen production. DFT is a powerful computational tool that enables the study of electronic structures, reaction kinetics, and reaction mechanisms at the atomic and molecular levels. By employing DFT calculations, researchers can gain a detailed understanding of the energetics and kinetics of various PEC reactions occurring at the electrode‐electrolyte interface. This knowledge can aid in the design and optimization of efficient photoelectrodes and catalysts, leading to improved performance and stability of the PEC system. By combining DFT simulations with experimental investigations, researchers can obtain a comprehensive understanding of the factors influencing the degradation of PEC systems, enabling the design of strategies to mitigate or prevent degradation altogether. Furthermore, DFT can assist in the screening and discovery of new materials with enhanced properties for photoelectrochemical green hydrogen production. Through virtual screening and high‐throughput calculations, DFT can identify promising candidate materials for photoelectrodes, catalysts, and protective coatings, significantly accelerating the material discovery process. This can lead to the development of novel materials with superior performance, efficiency, and stability, ultimately facilitating the industrial implementation of photoelectrochemical green hydrogen production.

Artificial intelligence (AI) and machine learning (ML) can play a crucial role in enhancing the practicality and comprehension of data obtained from PEC green hydrogen production, especially when combined with DFT calculations. PEC green hydrogen production involves intricate processes and interactions between materials, light, and electrochemical reactions. Theoretical data obtained through DFT calculations can provide insights into the electronic structure, band alignment, and reaction kinetics of various materials used in PEC cells. However, interpreting and comprehending such data can be challenging for researchers due to its complexity.

AI and ML techniques can aid in simplifying and extracting meaningful information from this complex data and can significantly speed up the material discovery process. By training ML models on large databases of DFT‐calculated properties, the models can learn to predict material properties and performance without performing computationally expensive calculations for each candidate. This enables researchers to efficiently identify promising materials for PEC applications, reducing the need for extensive experimental validation.

PEC green hydrogen production involves multiple interconnected components, including photoelectrodes, catalysts, electrolytes, and cell architectures. Optimizing these systems for enhanced efficiency and stability is a complex task that often requires iterative experimental and theoretical investigations. AI and ML can facilitate the optimization process by creating surrogate models that capture the relationships between input parameters and PEC performance metrics. By training these models on a combination of theoretical data and experimental results, researchers can explore the parameter space more efficiently and identify optimal system configurations. This can help accelerate the development of practical PEC systems with improved performance.

## Conflict of Interest

The authors declare no conflict of interest.

## References

[1] R. Schlögl , Green Chem. 2021, 23, 1584.

[2] M. Isaacs , J. Garcia‐Navarro , W.‐J. Ong , P. Jiménez‐Calvo , Global Challenges 2023, 7, 2200165. 36910466 10.1002/gch2.202200165 PMC10000254

[3] H. Song , S. Luo , H. Huang , B. Deng , J. Ye , ACS Energy Lett. 2022, 7, 1043.

[4] N. I. Badea , Energies 2021, 14, 5783.

[5] H. Menski , Focus on Hydrogen: Japan's energy strategy for hydrogen and ammonia. Focus on Hydrogen: Clifford Chance 2022.

[6] L. J. Gregor Erbach , Eur. Parliamentary Res. Service 2021.

[7] U.S. National Clean Hydrogen Strategy and Roadmap. U.S. Department of Energy (DOE) 2023.

[8] G. A. Reigstad , P. Coussy , J. Straus , C. Bordin , S. Jaehnert , SINTEF Rapport;: SINTEF Energi AS, 2019.

[9] M. Younas , S. Shafique , A. Hafeez , F. Javed , F. Rehman , Fuel 2022, 316, 123317.

[10] Y. Guo , Q. Zhou , X. Chen , Y. Fu , S. Lan , M. Zhu , Y. Du , J. Mater. Sci. Technol. 2022, 119, 53.

[11] C. Iffelsberger , S. Ng , M. Pumera , Chem. Eng. J. 2022, 446, 136995.

[12] J.‐W. Lee , K.‐H. Cho , J.‐S. Yoon , Y.‐M. Kim , Y.‐M. Sung , J. Mater. Chem. A 2021, 9, 21576.

[13] J. Joy , J. Mathew , S. C. George , Int. J. Hydrogen Energy 2018, 43, 4804.

[14] B. D. James , G. N. Baum , J. Perez , K. N. Baum , Technoeconomic Analysis of Photoelectrochemical (PEC) Hydrogen Production. Directed Technologies INC 2009.

[15] A. Grimm , W. A. de Jong , G. J. Kramer , Int. J. Hydrogen Energy 2020, 45, 22545.

[16] E. Kieckens , Spain starts producing renewable hydrogen through photoeletrocatalysis, https://innovationorigins.com/en/spain‐starts‐producing‐renewable‐hydrogen‐through‐photoeletrocatalysis/, (accessed: August 2021).

[17] S. Matalucci , The Hydrogen Stream: South Korean scientists achieve photocurrent density of 19.8 mA cm−2 in PEC cell for water splitting, https://www.pv‐magazine.com/2022/10/07/the‐hydrogen‐stream‐south‐korean‐scientists‐achieve‐photocurrent‐density‐of‐19‐8%E2%80%89ma%E2%80%89cm%E2%88%922‐in‐pec‐cell‐for‐water‐splitting/, (accessed: October 2022).

[18] J. Kiwi , M. Graetzel , J. Am. Chem. Soc. 1979, 101, 7214.

[19] A. Fujishima , K. Honda , Nature 1972, 238, 37. 12635268 10.1038/238037a0

[20] V. S. Vyas , V. W.‐h Lau , B. V. Lotsch , Chem. Mater. 2016, 28, 5191.

[21] S. U. M. Khan , R. C. Kainthla , J. O. M. Bockris , Int. J. Hydrogen Energy 1988, 13, 225.

[22] C. Gomes Silva , I. Luz , F. X. Llabrés i Xamena , A. Corma , H. García , Chemistry 2010, 16, 11133. 20687143 10.1002/chem.200903526

[23] N. Arai , N. Saito , H. Nishiyama , K. Domen , H. Kobayashi , K. Sato , Y. Inoue , Catal. Today 2007, 129, 407.

[24] T. Yokoi , J. Sakuma , K. Maeda , K. Domen , T. Tatsumi , J. N. Kondo , Phys. Chem. Chem. Phys. 2011, 13, 2563. 21221444 10.1039/c0cp02141e

[25] T. Takata , A. Tanaka , M. Hara , J. N. Kondo , K. Domen , Catal. Today 1998, 44, 17.

[26] M. A. Bañares , M. O. Guerrero‐Pérez , J. L. G. Fierro , G. G. Cortez , J. Mater. Chem. 2002, 12, 3337.

[27] A. Braun , K. Sivula , D. K. Bora , J. Zhu , L. Zhang , M. Grätzel , J. Guo , E. C. Constable , J. Phys. Chem. C 2012, 116, 16870.

[28] W. Yang , T. Moehl , E. Service , S. D. Tilley , Adv. Energy Mater. 2021, 11, 2003569.

[29] S. Guo , Y. Li , S. Tang , Y. Zhang , X. Li , A. J. Sobrido , M.‐M. Titirici , B. Wei , Adv. Funct. Mater. 2020, 30, 2003035.

[30] M. Favaro , J. Yang , S. Nappini , E. Magnano , F. M. Toma , E. J. Crumlin , J. Yano , I. D. Sharp , J. Am. Chem. Soc. 2017, 139, 8960. 28598604 10.1021/jacs.7b03211

[31] O. Zandi , T. W. Hamann , Nat. Chem. 2016, 8, 778. 27442283 10.1038/nchem.2557

[32] T. Baran , M. Fracchia , A. Vertova , E. Achilli , A. Naldoni , F. Malara , G. Rossi , S. Rondinini , P. Ghigna , A. Minguzzi , F. D'Acapito , Electrochim. Acta 2016, 207, 16.

[33] N. Han , P. Liu , J. Jiang , L. Ai , Z. Shao , S. Liu , J. Mater. Chem. A 2018, 6, 19912.

[34] F. Sordello , P. Calza , C. Minero , S. Malato , M. Minella , Catalysts 2022, 12, 1572.

[35] D. C. Bookbinder , J. A. Bruce , R. N. Dominey , N. S. Lewis , M. S. Wrighton , Proc. Natl. Acad. Sci. USA 1980, 77, 6280. 16592907 10.1073/pnas.77.11.6280 PMC350266

[36] K. Shankar , J. I. Basham , N. K. Allam , O. K. Varghese , G. K. Mor , X. Feng , M. Paulose , J. A. Seabold , K.‐S. Choi , C. A. Grimes , J. Phys. Chem. C 2009, 113, 6327.

[37] J. Xing , J. F. Chen , Y. H. Li , W. T. Yuan , Y. Zhou , L. R. Zheng , H. F. Wang , P. Hu , Y. Wang , H. J. Zhao , Y. Wang , H. G. Yang , Chemistry 2014, 20, 2138. 24403011 10.1002/chem.201303366

[38] G. Yang , S. Li , X. Wang , B. Ding , Y. Li , H. Lin , D. Tang , X. Ren , Q. Wang , S. Luo , J. Ye , Appl. Catal., B 2021, 297, 120268.

[39] M. A. Marwat , M. Humayun , M. W. Afridi , H. Zhang , M. R. Abdul Karim , M. Ashtar , M. Usman , S. Waqar , H. Ullah , C. Wang , W. Luo , ACS Appl. Energy Mater. 2021, 4, 12007.

[40] L. Mascaretti , A. Dutta , S. Kment , V. M. Shalaev , A. Boltasseva , R. Zboril , A. Naldoni , Adv. Mater. 2019, 31, 1805513. 10.1002/adma.201805513 30773753

[41] F. E. Osterloh , Chem. Soc. Rev. 2013, 42, 2294. 23072874 10.1039/c2cs35266d

[42] K. Sun , S. Shen , Y. Liang , P. E. Burrows , S. S. Mao , D. Wang , Chem. Rev. 2014, 114, 8662. 25084474 10.1021/cr300459q

[43] K. Iwashina , A. Iwase , Y. H. Ng , R. Amal , A. Kudo , J. Am. Chem. Soc. 2015, 137, 604. 25551584 10.1021/ja511615s

[44] J. Sato , N. Saito , Y. Yamada , K. Maeda , T. Takata , J. N. Kondo , M. Hara , H. Kobayashi , K. Domen , Y. Inoue , J. Am. Chem. Soc. 2005, 127, 4150. 15783179 10.1021/ja042973v

[45] J. Tournet , Y. Lee , S. K. Karuturi , H. H. Tan , C. Jagadish , ACS Energy Lett. 2020, 5, 611.

[46] D. Duonghong , E. Borgarello , M. Graetzel , J. Am. Chem. Soc. 1981, 103, 4685.

[47] H. Jung , Y. Kwon , Y. Kim , H. Ahn , H. Ahn , Y. Wy , S. W. Han , Nano Lett. 2023, 23, 1774. 36802375 10.1021/acs.nanolett.2c04544

[48] T. Chen , Q. Ding , X. Wang , Z. Feng , C. Li , J. Phys. Chem. Lett. 2021, 12, 6029. 34165306 10.1021/acs.jpclett.1c01621

[49] B. M. Weckhuysen , Chem. Commun. 2002, 10.1039/B107686H. 12120361

[50] M. Fracchia , V. Cristino , A. Vertova , S. Rondinini , S. Caramori , P. Ghigna , A. Minguzzi , Electrochim. Acta 2019, 320, 134561.

[51] F. Malara , M. Fracchia , H. Kmentová , R. Psaro , A. Vertova , D. Oliveira de Souza , G. Aquilanti , L. Olivi , P. Ghigna , A. Minguzzi , A. Naldoni , ACS Catal. 2020, 10, 10476.

[52] L. Li , J. Yang , H. Ali‐Löytty , T.‐C. Weng , F. M. Toma , D. Sokaras , I. D. Sharp , A. Nilsson , ACS Appl. Energy Mater. 2019, 2, 1371.

[53] N. Sivasankar , W. W. Weare , H. Frei , J. Am. Chem. Soc. 2011, 133, 12976. 21770440 10.1021/ja205300a

[54] Y. Zhang , H. Zhang , A. Liu , C. Chen , W. Song , J. Zhao , J. Am. Chem. Soc. 2018, 140, 3264. 29455534 10.1021/jacs.7b10979

[55] Q.‐Y. Wang , Y.‐Y. Chen , R.‐K. Ye , Q. Liu , H.‐Y. Chen , H. Yang , M.‐Y. Li , J.‐Q. Hu , P.‐P. Fang , Anal. Chem. 2021, 93, 15517. 34726908 10.1021/acs.analchem.1c03666

[56] A. Venugopal , R. Kas , K. Hau , W. A. Smith , J. Am. Chem. Soc. 2021, 143, 18581. 34726398 10.1021/jacs.1c08245 PMC8587602

[57] T. Moehl , W. Cui , R. Wick‐Joliat , S. D. Tilley , Sustainable Energy Fuels 2019, 3, 2067.

[58] W. Cui , T. Moehl , S. Siol , S. D. Tilley , Sustainable Energy Fuels 2019, 3, 3085.

[59] G. G. Bessegato , T. T. Guaraldo , J. F. de Brito , M. F. Brugnera , M. V. B. Zanoni , Electrocatalysis 2015, 6, 415.

[60] P. Zhou , I. A. Navid , Y. Ma , Y. Xiao , P. Wang , Z. Ye , B. Zhou , K. Sun , Z. Mi , Nature 2023, 613, 66. 36600066 10.1038/s41586-022-05399-1

[61] S. Chu , S. Vanka , Y. Wang , J. Gim , Y. Wang , Y.‐H. Ra , R. Hovden , H. Guo , I. Shih , Z. Mi , ACS Energy Lett. 2018, 3, 307.

[62] P. Alulema‐Pullupaxi , P. J. Espinoza‐Montero , C. Sigcha‐Pallo , R. Vargas , L. Fernández , J. M. Peralta‐Hernández , J. L. Paz , Chemosphere 2021, 281, 130821. 34000653 10.1016/j.chemosphere.2021.130821

[63] Z. Yin , R. Fan , G. Huang , M. Shen , Chem. Commun. 2018, 54, 543. 10.1039/c7cc08409a 29292435

[64] D. Kang , J. L. Young , H. Lim , W. E. Klein , H. Chen , Y. Xi , B. Gai , T. G. Deutsch , J. Yoon , Nat. Energy 2017, 2, 17043.

[65] Y. Wang , J. Schwartz , J. Gim , R. Hovden , Z. Mi , ACS Energy Lett. 2019, 4, 1541.

[66] X. Zhang , H. Cui , M. Humayun , Y. Qu , N. Fan , X. Sun , L. Jing , Sci. Rep. 2016, 6, 21430. 26906953 10.1038/srep21430 PMC4764924

[67] M. Kumar , B. Meena , P. Subramanyam , D. Suryakala , C. Subrahmanyam , NPG Asia Mater. 2022, 14, 88.

[68] C. Sharma , D. Pooja , A. Thakur , Y. S. Negi , ECS Adv. 2022, 1, 030501.

[69] Y. Miao , M. Shao , Chin. J. Catal. 2022, 43, 595.

[70] H. H. Mohamed , in Sustainable Materials and Green Processing for Energy Conversion, (Eds: K. Y. Cheong , A. Apblett ), Elsevier, Amsterdam 2022, pp. 169–212.

[71] Z. Li , W. Luo , M. Zhang , J. Feng , Z. Zou , Energy Environ. Sci. 2013, 6, 347.

[72] S. Hu , C. Xiang , S. Haussener , A. D. Berger , N. S. Lewis , Energy Environ. Sci. 2013, 6, 2984.

[73] T. Yao , X. An , H. Han , J. Q. Chen , C. Li , Adv. Energy Mater. 2018, 8, 1800210.

[74] W. Yang , R. R. Prabhakar , J. Tan , S. D. Tilley , J. Moon , Chem. Soc. Rev. 2019, 48, 4979. 31483417 10.1039/c8cs00997j

[75] C. Ros , T. Andreu , J. R. Morante , J. Mater. Chem. A 2020, 8, 10625.

[76] J. H. Kim , D. Hansora , P. Sharma , J.‐W. Jang , J. S. Lee , Chem. Soc. Rev. 2019, 48, 1908. 30855624 10.1039/c8cs00699g

[77] B. Moss , O. Babacan , A. Kafizas , A. Hankin , Adv. Energy Mater. 2021, 11, 2003286.

[78] S. Chu , W. Li , Y. Yan , T. Hamann , I. Shih , D. Wang , Z. Mi , Nano Futures 2017, 1, 022001.

[79] S. S. Mao , S. Shen , L. Guo , Prog. Nat. Sci.: Mater. Int. 2012, 22, 522.

[80] S. Palmas , L. Mais , M. Mascia , A. Vacca , Curr. Opin. Electrochem. 2021, 28, 100699.

[81] S. S. Shinde , R. A. Bansode , C. H. Bhosale , K. Y. Rajpure , J. Semicond. 2011, 32, 013001.

[82] Z. Luo , T. Wang , J. Gong , Chem. Soc. Rev. 2019, 48, 2158. 30601502 10.1039/c8cs00638e

[83] L. M. Peter , Electroanalysis 2015, 27, 864.

[84] O. J. Alley , K. Wyatt , M. A. Steiner , G. Liu , T. Kistler , G. Zeng , D. M. Larson , J. K. Cooper , J. L. Young , T. G. Deutsch , F. M. Toma , Front. Energy Res. 2022, 10, 884364.

[85] H. Dotan , N. Mathews , T. Hisatomi , M. Grätzel , A. Rothschild , J. Phys. Chem. Lett. 2014, 5, 3330. 26278440 10.1021/jz501716g

[86] Z. Chen , T. F. Jaramillo , T. G. Deutsch , A. Kleiman‐Shwarsctein , A. J. Forman , N. Gaillard , R. Garland , K. Takanabe , C. Heske , M. Sunkara , E. W. McFarland , K. Domen , E. L. Miller , J. A. Turner , H. N. Dinh , J. Mater. Res. 2010, 25, 3.

[87] S. Vanka , G. Zeng , T. G. Deutsch , F. M. Toma , Z. Mi , Front. Energy Res. 2022, 10, 840140.

[88] S. Wang , G. Liu , L. Wang , Chem. Rev. 2019, 119, 5192. 30875200 10.1021/acs.chemrev.8b00584

[89] Q. Pan , A. Li , Y. Zhang , Y. Yang , C. Cheng , Adv. Sci. 2020, 7, 1902235. 10.1002/advs.201902235 PMC7001624 32042560

[90] J. Fu , Z. Fan , M. Nakabayashi , H. Ju , N. Pastukhova , Y. Xiao , C. Feng , N. Shibata , K. Domen , Y. Li , Nat. Commun. 2022, 13, 729. 35132086 10.1038/s41467-022-28415-4 PMC8821563

[91] M. A. Hassan , M.‐W. Kim , M. A. Johar , A. Waseem , M.‐K. Kwon , S.‐W. Ryu , Sci. Rep. 2019, 9, 20141. 31882920 10.1038/s41598-019-56807-y PMC6934777

[92] B. AlOtaibi , S. Fan , S. Vanka , M. G. Kibria , Z. Mi , Nano Lett. 2015, 15, 6821. 26360182 10.1021/acs.nanolett.5b02671

[93] M. K. Mohanta , T. K. Sahu , S. Bhowmick , M. Qureshi , Electrochim. Acta 2022, 415, 140269.

[94] B. Liu , S. Wang , S. Feng , H. Li , L. Yang , T. Wang , J. Gong , Adv. Funct. Mater. 2021, 31, 2007222.

[95] H. Geng , P. Ying , K. Li , Y. Zhao , X. Gu , Appl. Surf. Sci. 2021, 563, 150289.

[96] F. Li , Y. Li , Q. Zhuo , D. Zhou , Y. Zhao , Z. Zhao , X. Wu , Y. Shan , L. Sun , ACS Appl. Mater. Interfaces 2020, 12, 11479. 32056436 10.1021/acsami.9b19418

[97] H. Li , T. Wang , S. Liu , Z. Luo , L. Li , H. Wang , Z.‐J. Zhao , J. Gong , Angew. Chem., Int. Ed. 2021, 60, 4034. 10.1002/anie.202014538 33185337

[98] Y. Li , Q. Mei , Z. Liu , X. Hu , Z. Zhou , J. Huang , B. Bai , H. Liu , F. Ding , Q. Wang , Appl. Catal., B 2022, 304, 120995.

[99] Y. Kuang , Q. Jia , G. Ma , T. Hisatomi , T. Minegishi , H. Nishiyama , M. Nakabayashi , N. Shibata , T. Yamada , A. Kudo , K. Domen , Nat. Energy 2016, 2, 16191.

[100] Y. Xiao , C. Feng , J. Fu , F. Wang , C. Li , V. F. Kunzelmann , C.‐M. Jiang , M. Nakabayashi , N. Shibata , I. D. Sharp , K. Domen , Y. Li , Nat. Catal. 2020, 3, 932.

[101] Q. Li , M. Zheng , B. Zhang , C. Zhu , F. Wang , J. Song , M. Zhong , L. Ma , W. Shen , Nanotechnology 2016, 27, 075704. 26775672 10.1088/0957-4484/27/7/075704

[102] A. Syed Asim , T. Ahmad , Int. J. Hydrogen Energy 2023, 48‐58, 22044.

[103] A. Syed Asim , T. Ahmad , Inorg. Chem. 2024, 63, 304. 38146688 10.1021/acs.inorgchem.3c03176

[104] H. Khan , S. E. Lofland , J. Ahmed , K. V. Ramanujachary , T. Ahmad , Int. J. Hydrogen Energy 2024, 58, 717.

[105] H. Khan , S. E. Lofland , J. Ahmed , K. V. Ramanujachary , T. Ahmad , Int. J. Hydrogen Energy 2024, 58, 954.

[106] S. A. Ali , T. Ahmad , Mater. Today Chem. 2023, 29, 10138.

[107] H. Khan , I. H. Lone , S. E. Lofland , K. V. Ramanujachary , T. Ahmad , Int. J. Hydrogen Energy 2023, 48‐14, 5493.

[108] Y. Huang , Y. Chen , L. Deng , Y. Zhu , Y. Huang , Appl. Phys. Lett. 2021, 119, 043903.

[109] S. Li , H. Lin , G. Yang , X. Ren , S. Luo , X. Wang , Z. Chang , J. Ye , Appl. Catal., B 2022, 304, 120954.

[110] H.‐C. Fu , P. Varadhan , M.‐L. Tsai , W. Li , Q. Ding , C.‐H. Lin , M. Bonifazi , A. Fratalocchi , S. Jin , J.‐H. He , Nano Energy 2020, 70, 104478.

[111] Z. Zhang , L. Zhang , M. N. Hedhili , H. Zhang , P. Wang , Nano Lett. 2013, 13, 14. 23205530 10.1021/nl3029202

[112] F. Nan , T. Cai , S. Ju , L. Fang , Appl. Phys. Lett. 2018, 112, 173902.

[113] J. Huang , D. Chu , K. Li , X. Li , A. Liu , C. Zhang , Y. Du , J. Phys. Chem. C 2018, 122, 260.

[114] J. J. Pietron , P. A. DeSario , J. Photonics Energy 2016, 7‐1, 012007.

[115] X. He , W. Tian , L. Yang , Z. Bai , L. Li , Small Methods 2024, 8‐2, 2300350. 10.1002/smtd.202300350 37330656

[116] J. Joy , J. Mathew , S. C. George , Int. J. Hydrogen Energy 2018, 43‐10, 4804.

[117] P. Subramanyam , B. Meena , V. Biju , H. Misawa , C. Subrahmanyam , J. Photochem. Photobiol., C 2022, 51, 100472.

[118] Z. Bielan , K. Siuzdak , Materials for Hydrogen Production, Conversion, and Storage, 2023, pp. 1–39.

[119] Z. Li , L. Luo , M. Li , W. Chen , Y. Liu , J. Yang , S.‐M. Xu , H. Zhou , L. Ma , M. Xu , X. Kong , H. Duan , Nat. Commun. 2021, 12, 6698. 34795245 10.1038/s41467-021-26997-z PMC8602285

[120] S. Mohajernia , S. Hejazi , A. Mazare , N. T. Nguyen , P. Schmuki , Chemistry 2017, 23, 12406. 28654181 10.1002/chem.201702245

[121] F. Yu , F. Li , T. Yao , J. Du , Y. Liang , Y. Wang , H. Han , L. Sun , ACS Catal. 2017, 7, 1868.

[122] S. He , C. Yan , X.‐Z. Chen , Z. Wang , T. Ouyang , M.‐L. Guo , Z.‐Q. Liu , Appl. Catal., B 2020, 276, 119138.

[123] M. Ge , J. Cai , J. Iocozzia , C. Cao , J. Huang , X. Zhang , J. Shen , S. Wang , S. Zhang , K.‐Q. Zhang , Y. Lai , Z. Lin , Int. J. Hydrogen Energy 2017, 42, 8418.

[124] M. Ni , M. K. H. Leung , D. Y. C. Leung , K. Sumathy , Renewable Sustainable Energy Rev. 2007, 11, 401.

[125] L. Shi , D. Benetti , F. Li , Q. Wei , F. Rosei , Appl. Catal., B 2020, 263, 118317.

[126] S. I. Park , S.‐M. Jung , J.‐Y. Kim , J. Yang , Materials 2022, 15, 6010.36079393

[127] Z. Dong , D. Ding , T. Li , C. Ning , Appl. Surf. Sci. 2019, 480, 219.

[128] D. Gogoi , A. Namdeo , A. K. Golder , N. R. Peela , Int. J. Hydrogen Energy 2020, 45, 2729.

[129] M. P. Kumar , R. Jagannathan , S. Ravichandran , Energy Fuels 2020, 34, 9030.

[130] H. Yin , Y. Wang , L. Ma , S. Zhang , B. Yang , R. Jiang , Chem. Eng. J. 2022, 431, 134124.

[131] W. Cui , H. Bai , J. Shang , F. Wang , D. Xu , J. Ding , W. Fan , W. Shi , Electrochim. Acta 2020, 349, 136383.

[132] H. S. Han , S. Shin , D. H. Kim , I. J. Park , J. S. Kim , P. S. Huang , J. K. Lee , I. S. Cho , X. Zheng , Energy Environ. Sci. 2018, 11, 1299.

[133] S. Lany , J. Phys.: Condens. Matter 2015, 27, 283203. 26126022 10.1088/0953-8984/27/28/283203

[134] J. H. Kim , J. S. Lee , Adv. Mater. 2019, 31, 1806938.

[135] X. Yao , X. Zhao , J. Hu , H. Xie , D. Wang , X. Cao , Z. Zhang , Y. Huang , Z. Chen , T. Sritharan , iScience 2019, 19, 976. 31522120 10.1016/j.isci.2019.08.037 PMC6744392

[136] D. K. Lee , K.‐S. Choi , Nat. Energy 2018, 3, 53.

[137] M. R. Nellist , J. Qiu , F. A. L. Laskowski , F. M. Toma , S. W. Boettcher , ACS Energy Lett. 2018, 3, 2286.

[138] D. A. Reddy , Y. Kim , H. S. Shim , K. A. J. Reddy , M. Gopannagari , D. Praveen Kumar , J. K. Song , T. K. Kim , ACS Appl. Energy Mater. 2020, 3, 4474.

[139] H. Pei , S. Xu , Y. Zhang , Y. Zhou , R. Li , T. Peng , Appl. Catal., B 2022, 318, 121865.

[140] F. M. Toma , J. K. Cooper , V. Kunzelmann , M. T. McDowell , J. Yu , D. M. Larson , N. J. Borys , C. Abelyan , J. W. Beeman , K. M. Yu , J. Yang , L. Chen , M. R. Shaner , J. Spurgeon , F. A. Houle , K. A. Persson , I. D. Sharp , Nat. Commun. 2016, 7, 12012. 27377305 10.1038/ncomms12012 PMC4935965

[141] H. Zhang , D. Li , W. J. Byun , X. Wang , T. J. Shin , H. Y. Jeong , H. Han , C. Li , J. S. Lee , Nat. Commun. 2020, 11, 4622. 32934221 10.1038/s41467-020-18484-8 PMC7493915

[142] J. Yin , J. Jin , H. Lin , Z. Yin , J. Li , M. Lu , L. Guo , P. Xi , Y. Tang , C.‐H. Yan , Adv. Sci. 2020, 7, 1903070. 10.1002/advs.201903070 PMC7237848 32440471

[143] S. Anantharaj , S. R. Ede , K. Sakthikumar , K. Karthick , S. Mishra , S. Kundu , ACS Catal. 2016, 6, 8069.

[144] J. Huang , Y. Jiang , T. An , M. Cao , J. Mater. Chem. A 2020, 8, 25465.

[145] Y. Zhao , S. Wei , K. Pan , Z. Dong , B. Zhang , H.‐H. Wu , Q. Zhang , J. Lin , H. Pang , Chem. Eng. J. 2021, 421, 129645.

[146] V.‐H. Nguyen , T. P. Nguyen , T.‐H. Le , D.‐V. N. Vo , D. L. T. Nguyen , Q. T. Trinh , I. T. Kim , Q. V. Le , J. Chem. Technol. Biotechnol. 2020, 95, 2597.

[147] X.‐C. Lu , Y.‐Z. Lu , C. Wang , Y. Cao , Rare Met. 2022, 41, 1142.

[148] F. Bozheyev , K. Ellmer , J. Mater. Chem. A 2022, 10, 9327.

[149] A. Jäger‐Waldau , M. C. Lux‐Steiner , E. Bucher , Solid State Phenom. 1994, 37‐38, 479.

[150] J. R. McKone , A. P. Pieterick , H. B. Gray , N. S. Lewis , J. Am. Chem. Soc. 2013, 135, 223. 23198831 10.1021/ja308581g

[151] S. Chandrasekaran , L. Yao , L. Deng , C. Bowen , Y. Zhang , S. Chen , Z. Lin , F. Peng , P. Zhang , Chem. Soc. Rev. 2019, 48, 4178. 31206105 10.1039/c8cs00664d

[152] Z. Yin , B. Chen , M. Bosman , X. Cao , J. Chen , B. Zheng , H. Zhang , Small 2014, 10, 3537. 24610819 10.1002/smll.201400124

[153] M. I. Zappia , G. Bianca , S. Bellani , M. Serri , L. Najafi , R. Oropesa‐Nuñez , B. Martín‐García , D. Bousa , D. Sedmidubský , V. Pellegrini , Z. Sofer , A. Cupolillo , F. Bonaccorso , Adv. Funct. Mater. 2020, 30, 1909572.

[154] F. M. Oliveira , J. Pastika , L. S. Pires , Z. Sofer , R. Gusmão , Adv. Mater. Interfaces 2021, 8, 2100294.

[155] G. Bianca , M. I. Zappia , S. Bellani , Z. Sofer , M. Serri , L. Najafi , R. Oropesa‐Nuñez , B. Martín‐García , T. Hartman , L. Leoncino , D. Sedmidubský , V. Pellegrini , G. Chiarello , F. Bonaccorso , ACS Appl. Mater. Interfaces 2020, 12, 48598. 32960559 10.1021/acsami.0c14201 PMC8011798

[156] M. Mohamed Abouelela , G. Kawamura , A. Matsuda , J. Energy Chem. 2022, 73, 189.

[157] S. Jin , ACS Energy Lett. 2017, 2, 1937.

[158] J. N. Hausmann , P. W. Menezes , Curr. Opin. Electrochem. 2022, 34, 100991.

[159] J.‐H. Kim , K. Kawashima , B. R. Wygant , O. Mabayoje , Y. Liu , J. H. Wang , C. B. Mullins , ACS Appl. Energy Mater. 2018, 1, 5145. 10.1021/acsami.8b00304 29608854

[160] O. Mabayoje , A. Shoola , B. R. Wygant , C. B. Mullins , ACS Energy Lett. 2016, 1, 195.

[161] Q. Liu , H. Lu , Z. Shi , F. Wu , J. Guo , K. Deng , L. Li , ACS Appl. Mater. Interfaces 2014, 6, 17200. 25225738 10.1021/am505015j

[162] Y. Wang , F. Zhang , M. Yang , Z. Wang , Y. Ren , J. Cui , Y. Zhao , J. Du , K. Li , W. Wang , D. J. Kang , Microporous Mesoporous Mater. 2019, 284, 403.

[163] S. Li , L. Meng , W. Tian , L. Li , Adv. Energy Mater. 2022, 12, 2200629.

[164] Y. Li , Z. Liu , J. Zhang , Z. Guo , Y. Xin , L. Zhao , J. Alloys Compd. 2019, 790, 493.

[165] H. Wang , Y. Xia , H. Li , X. Wang , Y. Yu , X. Jiao , D. Chen , Nat. Commun. 2020, 11, 3078. 32555382 10.1038/s41467-020-16800-w PMC7299993

[166] A. Mehtab , P. P. Ingole , J. Ahmed , Y. Mao , T. Ahmad , J. Phys. Chem. C 2024, 128, 85.

[167] A. Mehtab , T. Ahmad , ACS Catal. 2024, 14, 691.

[168] B. Fabre , G. Loget , Acc Mater Res 2023, 4, 133.

[169] S. Vanka , E. Arca , S. Cheng , K. Sun , G. A. Botton , G. Teeter , Z. Mi , Nano Lett. 2018, 18, 6530. 30216079 10.1021/acs.nanolett.8b03087

[170] Z. Liu , C. Li , Y. Xiao , F. Wang , Q. Yu , M. B. Faheem , T. Zhou , Y. Li , J. Phys. Chem. C 2020, 124, 2844.

[171] S. Fan , B. AlOtaibi , S. Y. Woo , Y. Wang , G. A. Botton , Z. Mi , Nano Lett. 2015, 15, 2721. 25811636 10.1021/acs.nanolett.5b00535

[172] M. A. Green , E. D. Dunlop , D. H. Levi , J. Hohl‐Ebinger , M. Yoshita , A. W. Y. Ho‐Baillie , Prog. Photovoltaics 2019, 27, 565.

[173] P. Varadhan , H.‐C. Fu , Y.‐C. Kao , R.‐H. Horng , J.‐H. He , Nat. Commun. 2019, 10, 5282. 31754117 10.1038/s41467-019-12977-x PMC6872648

[174] J. Gu , J. A. Aguiar , S. Ferrere , K. X. Steirer , Y. Yan , C. Xiao , J. L. Young , M. Al‐Jassim , N. R. Neale , J. A. Turner , Nat. Energy 2017, 2, 16192.

[175] M. A. Hassan , J.‐H. Kang , M. A. Johar , J.‐S. Ha , S.‐W. Ryu , Acta Mater. 2018, 146, 171.

[176] Z. Xu , S. Zhang , J. Liang , J. Lin , Y. Yu , R. Li , F. Gao , G. Li , J. Power Sources 2019, 419, 65.

[177] H. Li , B. Zhang , Z. Wang , P. Wang , Y. Liu , X. Zhang , X. Qin , Y. Dai , M.‐H. Whangbo , B. Huang , Sol. RRL 2018, 2, 1700243.

[178] W. Si , D. Pergolesi , F. Haydous , A. Fluri , A. Wokaun , T. Lippert , Phys. Chem. Chem. Phys. 2017, 19, 656. 10.1039/c6cp07253d 27918033

[179] R. S. Ningthoujam , N. S. Gajbhiye , Prog. Mater. Sci. 2015, 70, 50.

[180] A. M. Smith , S. Nie , Acc. Chem. Res. 2010, 43, 190. 19827808 10.1021/ar9001069 PMC2858563

[181] M. D. Sharma , C. Mahala , M. Basu , ACS Appl. Nano Mater. 2021, 4, 3013.

[182] B. Y. Kaplan , A. C. Kirlioglu , M. Alinezhadfar , M. A. Zabara , N. R. Mojarrad , B. Iskandarani , A. Yürüm , C. S. Ozkan , M. Ozkan , S. A. Gürsel , Chem Catal. 2023, 3, 100601.

[183] D. Klotz , D. S. Ellis , H. Dotan , A. Rothschild , Phys. Chem. Chem. Phys. 2016, 18, 23438. 27524381 10.1039/c6cp04683e PMC5310524

[184] M. R. Nellist , F. A. L. Laskowski , J. Qiu , H. Hajibabaei , K. Sivula , T. W. Hamann , S. W. Boettcher , Nat. Energy 2018, 3, 46.

[185] Y. AlSalka , S. Schwabe , J. Geweke , G. Ctistis , H. Wackerbarth , Energy Technol. 2023, 11, 2200788.

[186] L. Peter , Curr. Opin. Green Sustainable Chem. 2021, 31, 100505.

[187] J. Li , J. Gong , Energy Environ. Sci. 2020, 13, 3748.

[188] M. A. Soldatov , P. V. Medvedev , V. Roldugin , I. N. Novomlinskiy , I. Pankin , H. Su , Q. Liu , A. V. Soldatov , Nanomaterials 2022, 12, 839. 35269331 10.3390/nano12050839 PMC8912469

[189] M. Yoshida , T. Yomogida , T. Mineo , K. Nitta , K. Kato , T. Masuda , H. Nitani , H. Abe , S. Takakusagi , T. Uruga , K. Asakura , K. Uosaki , H. Kondoh , Chem. Commun. 2013, 49, 7848. 10.1039/c3cc43584a 23892561

[190] G. Segev , H. Dotan , D. S. Ellis , Y. Piekner , D. Klotz , J. W. Beeman , J. K. Cooper , D. A. Grave , I. D. Sharp , A. Rothschild , Joule 2018, 2, 210.

[191] A. Kafizas , X. Wang , S. R. Pendlebury , P. Barnes , M. Ling , C. Sotelo‐Vazquez , R. Quesada‐Cabrera , C. Li , I. P. Parkin , J. R. Durrant , J. Phys. Chem. A 2016, 120, 715. 26777898 10.1021/acs.jpca.5b11567

[192] J. Zhang , B. Zhu , L. Zhanga , J. Yu , Chem. Commun. 2023,59, 688. 10.1039/d2cc06300j 36598049

[193] J. Ma , T. J. Miao , J. Tang , Chem. Soc. Rev. 2022, 51, 5777. 35770623 10.1039/d1cs01164b

[194] M. Forster , D. W. F. Cheung , A. M. Gardner , A. J. Cowan , J. Chem. Phys. 2020, 153, 150901. 33092350 10.1063/5.0022138

[195] J. Ravensbergen , F. F. Abdi , J. H. Santen , R. N. Frese , B. Dam , R. Krol , J. T. M. Kennis , J. Phys. Chem. C 2014, 118, 27793.

[196] I. Grigioni , L. Ganzer , F. V. A. Camargo , B. Bozzini , G. Cerullo , E. Selli , ACS Energy Lett. 2019, 4, 2213.

[197] D. H. K. Murthy , H. Matsuzaki , Z. Wang , Y. Suzuki , T. Hisatomi , K. Seki , Y. Inoue , K. Domen , Chem. Sci. 2019,10, 5353. 31191893 10.1039/c9sc00217k PMC6540954

[198] C.‐L. Dong , L. Vayssieres , Chemistry 2018, 24, 18356. 30300939 10.1002/chem.201803936

[199] Z. Gong , Y. Yang , J. Energy Chem. 2018, 27, 1566.

[200] T.‐K. Sham , Adv. Mater. 2014, 26, 7896. 24861360 10.1002/adma.201304349

[201] S. R. Bare , T. Ressler , Advances in Catalysis, Academic Press, Cambridge 2009, pp. 339–465.

[202] Y.‐S. Liu , P.‐A. Glans , C.‐H. Chuang , M. Kapilashrami , J. Guo , J. Electron Spectrosc. Relat. Phenom. 2015, 200, 282.

[203] A. J. Bard , C. G. Zoski , Electroanalytical Chemistry, CRC Press, Boca Raton, 2017.

[204] E. Fabbri , D. F. Abbott , M. Nachtegaal , T. J. Schmidt , Curr. Opin. Electrochem. 2017, 5, 20.

[205] S. Yamazoe , Y. Hitomi , T. Shishido , T. Tanaka , J. Phys. Chem. C 2008, 112, 6869.

[206] Y. Takagi , H. Wang , Y. Uemura , T. Nakamura , L. Yu , O. Sekizawa , T. Uruga , M. Tada , G. Samjeské , Y. Iwasawa , T. Yokoyama , Phys. Chem. Chem. Phys. 2017, 19, 6013. 28184398 10.1039/c6cp06634h

[207] L. Vayssieres , T. Lindgren , L. Vayssieres , H. Wang , S.‐E. Lindquist , in Chemical Physics of Nanostructured Semiconductors, (Eds: A. I. Kokorin , D. W. Bahnemann ), VSP, Utrecht, The Netherlands, 2003, pp. 83–110.

[208] K. Sivula , F. Le Formal , M. Grätzel , ChemSusChem 2011, 4, 432. 21416621 10.1002/cssc.201000416

[209] Y. Liu , C. Wei , C. K. Ngaw , Y. Zhou , S. Sun , S. Xi , Y. Du , J. S. C. Loo , J. W. Ager , Z. J. Xu , ACS Appl. Energy Mater. 2018, 1, 814.

[210] K. Sun , F. H. Saadi , M. F. Lichterman , W. G. Hale , H.‐P. Wang , X. Zhou , N. T. Plymale , S. T. Omelchenko , J.‐H. He , K. M. Papadantonakis , B. S. Brunschwig , N. S. Lewis , Proc. Natl. Acad. Sci. USA 2015, 112, 3612. 25762067 10.1073/pnas.1423034112 PMC4378389

[211] N. Guijarro , M. S. Prévot , K. Sivula , Phys. Chem. Chem. Phys. 2015, 17, 15655. 26030025 10.1039/c5cp01992c

[212] F. Malara , F. Fabbri , M. Marelli , A. Naldoni , ACS Catal. 2016, 6, 3619.

[213] J. Vura‐Weis , C.‐M. Jiang , C. Liu , H. Gao , J. M. Lucas , F. M. F. de Groot , P. Yang , A. P. Alivisatos , S. R. Leone , J. Phys. Chem. Lett. 2013, 4, 3667.

[214] R. Liu , Z. Zheng , J. Spurgeon , X. Yang , Energy Environ. Sci. 2014, 7, 2504.

[215] K. M. H. Young , B. M. Klahr , O. Zandi , T. W. Hamann , Catal. Sci. Technol. 2013, 3, 1660.

[216] F. Le Formal , K. Sivula , M. Grätzel , J. Phys. Chem. C 2012, 116, 26707.

[217] B. Klahr , S. Gimenez , F. Fabregat‐Santiago , J. Bisquert , T. W. Hamann , Energy Environ. Sci. 2012, 5, 7626.

[218] F. A. L. Laskowski , M. R. Nellist , J. Qiu , S. W. Boettcher , J. Am. Chem. Soc. 2019, 141, 1394. 30537811 10.1021/jacs.8b09449

[219] M. Barroso , C. A. Mesa , S. R. Pendlebury , A. J. Cowan , T. Hisatomi , K. Sivula , M. Grätzel , D. R. Klug , J. R. Durrant , Proc. Natl. Acad. Sci. USA 2012, 109, 15640. 22802673 10.1073/pnas.1118326109 PMC3465443

[220] D. K. Zhong , M. Cornuz , K. Sivula , M. Grätzel , D. R. Gamelin , Energy Environ. Sci. 2011, 4, 1759.

[221] G. M. Carroll , D. R. Gamelin , J. Mater. Chem. A 2016, 4, 2986.

[222] Z. Wang , G. Liu , C. Ding , Z. Chen , F. Zhang , J. Shi , C. Li , J. Phys. Chem. C 2015, 119, 19607.

[223] S. D. Tilley , M. Cornuz , K. Sivula , M. Grätzel , Angew. Chem. 2010, 122, 6549. 10.1002/anie.201003110 20665613

[224] C. L. Farrow , D. K. Bediako , Y. Surendranath , D. G. Nocera , S. J. L. Billinge , J. Am. Chem. Soc. 2013, 135, 6403. 23547707 10.1021/ja401276f

[225] L. Xi , C. Schwanke , D. Zhou , D. Drevon , R. van de Krol , K. M. Lange , Dalton Trans. 2017, 46, 15719. 29095446 10.1039/c7dt02647a

[226] M. W. Kanan , J. Yano , Y. Surendranath , M. Dinca , V. K. Yachandra , D. G. Nocera , J. Am. Chem. Soc. 2010, 132, 13692. 20839862 10.1021/ja1023767

[227] S. U. M. Khan , M. Al‐Shahry , W. B. Ingler , Science 2002, 297, 2243. 12351783 10.1126/science.1075035

[228] X. Yang , A. Wolcott , G. Wang , A. Sobo , R. C. Fitzmorris , F. Qian , J. Z. Zhang , Y. Li , Nano Lett. 2009, 9, 2331. 19449878 10.1021/nl900772q

[229] Z. Zhang , C. Shao , X. Li , C. Wang , M. Zhang , Y. Liu , ACS Appl. Mater. Interfaces 2010, 2, 2915. 20936796 10.1021/am100618h

[230] Y.‐K. Hsu , Y.‐C. Chen , Y.‐G. Lin , ACS Appl. Mater. Interfaces 2015, 7, 14157. 26053274 10.1021/acsami.5b03921

[231] M. Law , L. E. Greene , J. C. Johnson , R. Saykally , P. Yang , Nat. Mater. 2005, 4, 455. 15895100 10.1038/nmat1387

[232] Y. R. Lu , Y. F. Wang , H. W. Chang , Y. C. Huang , J. L. Chen , C. L. Chen , Y. C. Lin , Y. G. Lin , W. F. Pong , T. Ohigashi , N. Kosugi , C. H. Kuo , W. C. Chou , C. L. Dong , Sol. Energy Mater. Sol. Cells 2020, 209, 110469.

[233] J. W. Chiou , S. C. Ray , H. M. Tsai , C. W. Pao , F. Z. Chien , W. F. Pong , M.‐H. Tsai , J. J. Wu , C. H. Tseng , C.‐H. Chen , J. F. Lee , J.‐H. Guo , Appl. Phys. Lett. 2007, 90, 192112.

[234] H. M. Chen , C. K. Chen , C. C. Lin , R.‐S. Liu , H. Yang , W.‐S. Chang , K.‐H. Chen , T.‐S. Chan , J.‐F. Lee , D. P. Tsai , J. Phys. Chem. C 2011, 115, 21971.

[235] J. Yang , J. K. Cooper , F. M. Toma , K. A. Walczak , M. Favaro , J. W. Beeman , L. H. Hess , C. Wang , C. Zhu , S. Gul , J. Yano , C. Kisielowski , A. Schwartzberg , I. D. Sharp , Nat. Mater. 2017, 16, 335. 27820814 10.1038/nmat4794

[236] M. F. Lichterman , K. Sun , S. Hu , X. Zhou , M. T. McDowell , M. R. Shaner , M. H. Richter , E. J. Crumlin , A. I. Carim , F. H. Saadi , B. S. Brunschwig , N. S. Lewis , Catal. Today 2016, 262, 11.

[237] N. Danilovic , R. Subbaraman , K. C. Chang , S. H. Chang , Y. Kang , J. Snyder , A. P. Paulikas , D. Strmcnik , Y. T. Kim , D. Myers , V. R. Stamenkovic , N. M. Markovic , Angew. Chem., Int. Ed. 2014, 53, 14016. 10.1002/anie.201406455 25297010

[238] J. Rossmeisl , Z.‐W. Qu , H. Zhu , G.‐J. Kroes , J. K. Nørskov , J. Electroanal. Chem. 2007, 607, 83.

[239] J. Deng , Q. Zhang , X. Lv , D. Zhang , H. Xu , D. Ma , J. Zhong , ACS Energy Lett. 2020, 5, 975.

[240] M. Zhang , M. de Respinis , H. Frei , Nat. Chem. 2014, 6, 362. 24651205 10.1038/nchem.1874

[241] Y. Cao , D. Liu , X. Ni , X. Meng , Y. Zhou , Z. Sun , Y. Kuang , ACS Appl. Energy Mater. 2020, 3, 6334.

[242] Laboratory TNRE. News Release: Efficiency and Stability Best‐Practices Proposed for Solar Water‐Splitting To Make Hydrogen NREL, Berkeley Lab Offer Ideal Methods To Provide Confidence in Comparing Measurements, https://www.nrel.gov/news/press/2022/efficiency‐and‐stability‐best‐practices‐proposed‐for‐solar‐water‐splitting‐to‐make‐hydrogen.html (accessed: October 2022).

[243] J. Ke , F. He , H. Wu , S. Lyu , J. Liu , B. Yang , Z. Li , Q. Zhang , J. Chen , L. Lei , Y. Hou , K. Ostrikov , Nano‐Micro Lett. 2020, 13, 24. 10.1007/s40820-020-00545-8 PMC8187525 34138209

[244] X. Xie , R. Wang , Y. Ma , J. Chen , Q. Cui , Z. Shi , Z. Li , C. Xu , ACS Appl. Nano Mater. 2022, 5, 11150.

[245] D. Zhang , H.‐H. Cho , J.‐H. Yum , M. Mensi , K. Sivula , Adv. Energy Mater. 2022, 12, 2202363.

[246] L. Steier , S. Holliday , J. Mater. Chem. A 2018, 6, 21809.

[247] L. Yao , A. Rahmanudin , N. Guijarro , K. Sivula , Adv. Energy Mater. 2018, 8, 1802585.

[248] S. Otep , T. Michinobu , Q. Zhang , Sol. RRL 2020, 4, 1900395.

